# Photoacoustic imaging: An emerging tool for intraoperative margin assessment in breast-conserving surgery

**DOI:** 10.1016/j.pacs.2025.100788

**Published:** 2025-12-09

**Authors:** Zhijie Luo, Yiqiong Zheng, Ruixi Sun, Wenye Gong, Jiayu Wang, Guangwei Chen, Ye Zhang, Runqi Zhao, Daohuai Jiang, Fei Gao, Xiru Li

**Affiliations:** a PLA General Hospital (PLA Medical School), Beijing 100853, China; b Department of General Surgery, The First Medical Center of PLA General Hospital, Beijing 100853, China; c School of Biomedical Engineering, Division of Life Sciences and Medicine, University of Science and Technology of China, Hefei, Anhui 230026, China; d Hybrid Imaging System Laboratory, Suzhou Institute for Advanced Research, University of Science and Technology of China, Suzhou, Jiangsu 215123, China; e School of Information Science and Technology, ShanghaiTech University, Shanghai 201210, China; f Civil Aviation Medical Center Physical Examination and Appraisal Institute, Beijing 100123, China; g School of Medicine, Nankai University, Tianjin 300071, China; h Key Laboratory of Optoelectronic Science and Technology for Medicine, Ministry of Education, Fujian Provincial Key Laboratory for Photonics Technology, Fujian Normal University, Fuzhou, Fujian 350117, China; i School of Engineering Science, University of Science and Technology of China, Hefei, Anhui 230026, China

**Keywords:** Photoacoustic imaging, Breast cancer, Breast-conserving surgery, Tumor margin assessment

## Abstract

Photoacoustic Imaging (PAI) synergizes light's optical contrast with ultrasound's penetration depth via the photoacoustic effect. Breast cancer remains a global challenge, particular demanding precise intraoperative tumor demarcation during breast-conserving surgery (BCS). PAI has the potential to address this need by enabling boundary delineation, promoting complete resection and healthy tissue preservation. This review summarizes breast cancer epidemiology and BCS's clinical demands, highlighting PAI's unique advantages for intraoperative use. PAI can dynamically monitor cellular/tissue morphology, blood oxygen saturation, vasculature, and tumor-associated calcifications, generating high-contrast tumor margin information. This real-time feedback enhances surgical precision, reduces recurrence rates, and improves breast aesthetics and patient quality of life. Despite translational challenges, PAI is poised to become a revolutionary tool for optimizing BCS outcomes.

## 1 Introduction

### 1.1 The global burden of breast cancer and the shift toward quality of life

Breast cancer, as the most prevalent malignancy among women globally, has shown a significant upward trend in incidence, severely threatening female patients' health and quality of life. According to the World Health Organization (WHO), there were 2.3 million new breast cancer cases worldwide in 2020. Projections indicate this will exceed 3 million by 2040, with mortality reaching 1 million [1]. Notably, breast cancer incidence is increasingly affecting younger populations. In China, 14.6 % of patients are diagnosed under the age of 40, a proportion higher than in Western countries [2]. Despite rising incidence rates, mortality has declined steadily, with the 5-year overall survival rate for breast cancer patients in the United States reaching 91.2 % and 99.6 % for early-stage patients without lymph node metastasis. With prolonged survival, postoperative quality-of-life management and minimally invasive surgical techniques have become clinical priorities, particularly for younger patients who prioritize breast aesthetics.

### 1.2 Breast-conserving surgery and the critical challenge of margin assessment

In therapeutic strategies, breast-conserving surgery (BCS) combined with radiotherapy has emerged as the standard treatment for early-stage breast cancer due to its oncological safety and aesthetic benefits [3]. This approach is primarily indicated for clinical stage I-II (T1/T2 tumors) patients meeting specific criteria, including tumor size ≤ 5 cm, unifocal or quadrant-limited multifocal lesions, and absence of nipple-areolar complex invasion. For selected clinical stage III non-inflammatory breast cancer patients achieving tumor downstaging to R0 resection via neoadjuvant therapy, BCS may also be cautiously considered [4]. Studies confirm that BCS with radiotherapy achieves a 5-year local recurrence rate of 2 %-5 %, comparable to mastectomy, while significantly improving patients' quality of life and psychological well-being [5]. Over 60 % of breast cancer patients in the United States undergo BCS [6]. In China, its adoption rate has steadily increased with the promotion of Multidisciplinary diagnosis and treatment (MDT) models and improved awareness of BCS safety.

However, the success of BCS hinges entirely on a single, critical technical factor: achieving negative resection margins. International guidelines mandate ink-negative margins for invasive ductal carcinoma (IDC) and margins > 2 mm for ductal carcinoma in situ (DCIS) to minimize local recurrence risk [7], [8]. Precisely defining and excising the tumor boundary is the cornerstone of balancing oncological safety with the preservation of healthy tissue for optimal cosmetic results [9], [10], [11].

**Fig. 1:**
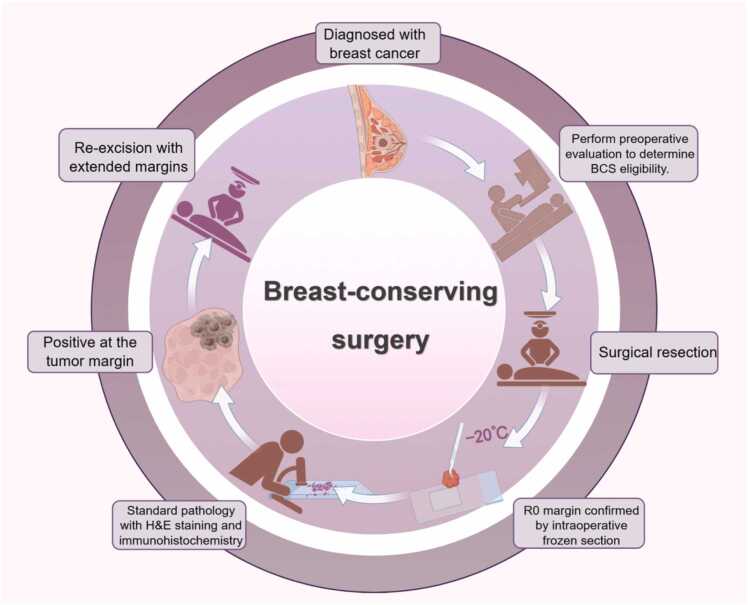
Management Flow Chart for Positive Margins in Breast-Conserving Surgery (BCS).

In clinical practice, margin assessment relies heavily on intraoperative palpation, visual inspection of tissue morphology/color, and preoperative imaging. After obtaining the initial information from the above assessments, surgeons often adopt a safety-first approach by extending resection margins by 0.5–1.0 cm, sacrificing excessive healthy tissue and compromising breast aesthetics to achieve R0 resection, i.e., microscopically negative margins [12], [13]. This subjective approach often leads to variable resection widths (from ink-negative to >10 mm) [14], [15], [16], with 20–40 % of BCS cases resulting in positive margins [7]. For positive margins, re-excision is recommended, but conversion to mastectomy becomes necessary if re-excision compromises aesthetics or fails to achieve negativity [7](Fig. 1). Reoperations impose additional psychological stress, prolonged recovery, increased costs, and higher complication risks [17].

After the tumor is cut off, the most commonly used method for intraoperative margin assessment remains frozen section (FS) analysis, including the tumor margin method and cavity margin method [18]. Based on the NSABP B-06 trial criteria [19], the tumor margin method involves ink staining of six specimen surfaces to determine tumor-to-ink distance. The cavity margin method reduces false-negative rates by excising additional peri-cavity tissues, which increases the risk of tissue damage [18]. In FS, tissues are rapidly frozen, sectioned, and microscopically examined [20], [21]. Pathologists evaluate cellular morphology, nuclear atypia, and architectural features to characterize tumor type. Postoperative paraffin-embedded sectioning, which is the gold standard, supplemented by immunohistochemical (IHC) staining (e.g., estrogen receptor, HER2, Ki-67), provides a definitive report on the tumor nature, grade, and metastasis status [22].

FS analysis, with a reported overall accuracy of > 95 % to paraffin-embedded sections, has been a benchmark for intraoperative margin assessment [20]. However, this high accuracy masks a critical weakness. Large-scale clinical evidence reveals a sensitivity of only 78–81 % compared to a specificity of 97–98 % [23], [24], [25]. This profile indicates that FS is highly reliable for confirming negative margins, but it fails to identify a notable proportion of positive margins. Consequently, this moderate sensitivity, combined with its time-consuming nature (∼30 min per sample) and potential to damage tissue for subsequent analysis, limits its effectiveness in guiding real-time surgical decisions and contributes to avoidable reoperations. These evidence-based limitations highlight the necessity for improved alternatives.

Precise margin assessment is not merely a technical step but a pivotal strategy to preserve normal tissue and achieve optimal breast morphology. By leveraging advanced intraoperative technologies, surgeons can enhance both survival outcomes and patient satisfaction, fulfilling the core objectives of BCS.

### 1.3 Current imaging methods for evaluating margins

These limitations drive the need for more efficient and accurate intraoperative assessment technologies to overcome the drawbacks of traditional pathological methods:

#### 1.3.1 Intraoperative mammography (e.g., BioVision)

**Fig. 2:**
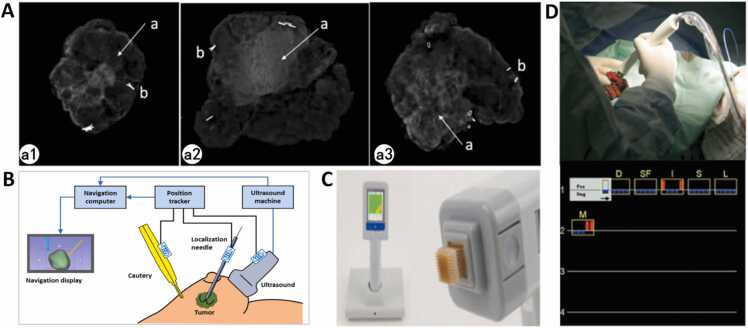
| A. ClearEdge Bioimpedance System [26] a1. Mass-type, a2. Calcification-type, a3. Mass with calcifications-type (a. Lesion, b. Titanium clip). B. Electromagnetic Navigation System (NaviKnife Schematic Diagram) [29]. C. ClearEdge System [30]. D. MarginProbe® Radiofrequency Spectroscopy System [32].

BioVision performs real-time imaging of resected specimens with a resolution of 48 μm (2–3 times higher than standard mammography), enabling enhanced visualization of microcalcifications or calcification clusters [26] (Fig. 2A). However, its sensitivity for non-calcified invasive foci remains low, and radiation exposure risks persist.

#### 1.3.2 Intraoperative ultrasound (IOUS)

IOUS improves resection accuracy for non-palpable lesions (e.g., DCIS, post-neoadjuvant residual tumors) through real-time boundary visualization. Studies demonstrate that IOUS reduces re-excision rates to 14 % [27]. Compared to palpation or wire-guided localization, IOUS decreases specimen volume and increases 1-year postoperative cosmetic satisfaction by 40 % [28]. Nevertheless, IOUS exhibits lower resolution than CT/MRI, poor DCIS detection, and limited differentiation of lesions with similar echogenicity.

#### 1.3.3 Electromagnetic navigation (e.g., NaviKnife System)

The NaviKnife system integrates preoperative MRI with intraoperative electromagnetic coordinates for real-time tumor boundary tracking (Fig. 2B), reducing specimen volume from 140.7 cm³ to 95.4 cm³. It is particularly useful in guiding nonpalpable tumor excision [29]. However, electromagnetic interference from surgical instruments and incompatibility with breast implants/metal prostheses restrict its clinical applicability.

#### 1.3.4 Radiofrequency spectroscopy (e.g., MarginProbe® RF Spectroscopy, ClearEdge Bioimpedance)

These technologies detect malignant cells via tissue bioimpedance. ClearEdge visualizes margin status based on dielectric properties of tissue, achieving a 91 % positive margin detection rate in a 45-case prospective trial [30] (Fig. 2C). The core principle of the MarginProbe**®** system is to distinguish between cancerous tissues and normal tissues based on the differences in membrane potentials, cell to cell connectivity, nuclear morphology, and vascularity [31]. It analyzes specimens within 3–5 min, reducing reoperation rates from 25.8 % to 10.9 % in early studies [32] (Fig. 2D). However, critical limitations persist: Ex vivo detection modes lose anatomical context due to tissue retraction-induced positioning errors (2–3 mm), preventing real-time resection boundary adjustments. ClearEdge suffers signal variability when contacting hemorrhagic tissue. Additionally, the device was designed with an emphasis on sensitivity to provide maximal detection of all positive margins. MarginProbe® exhibits a 53.6 % false-positive rate, which leads to excessive unnecessary extensive resections and removal of healthy tissue [31].

Therefore, to reduce positive margin rates and reoperation rates, there is a clinical imperative to develop advanced intraoperative margin assessment technologies capable of providing real-time, high-fidelity boundary evaluation during surgery. Such technologies must ensure complete tumor resection while minimizing the removal of healthy tissue, enhancing patient outcomes, and recovery trajectories Table 1.

**Table 1:** Comparative analysis of intraoperative margin assessment technologies for breast-conserving surgery.

**Parameter**	**Intraoperative Mammography**	**Intraoperative Ultrasound**	**Electromagnetic Navigation**	**Radiofrequency spectroscopy**	**Photoacoustic Imaging**
**Resolution**	48 μm (e.g., BioVision system) [26]	Approximately 2 mm [33]	Positional error and rotational error are approximately 0.90 mm and 0.31°, respectively [34].	Surface margins ≤ 1 mm in depth	PACT can achieve micrometer-level resolution; PMA can achieve nanometer-level resolution.
**Imaging Time**	Real-time imaging [26], [35]	Real-time imaging [33], [36]	Real-time imaging [29], [37]	All margin assessments are completed within 5 min [30], [32];The MarginProbe device is used on excised tissue immediately following excision (i.e., within 20 min).	Average preoperative/postoperative imaging time: 3 min; Sentinel lymph node assessment: up to 10 min; Total imaging time per patient: < 5 min (for MSOT Acuity Echo) [38].
**Sensitivity**	0.55 [35]	0.59 [36]	There are no clinical trial data available.	87.3 %（ClearEdge） [30]; 67 %（MarginProbe） [39]	A study achieved 100 % sensitivity. [38]
**Specificity**	0.85 [35]	0.81 [36]	There are no clinical trial data available.	75.6 %（ClearEdge） [30] 60–66.4 %（MarginProbe） [39]	There are no clinical trial data available.
**Impact on Surgical Time**	Intraoperative specimen mammography (ISM) reduced the average operation time by 42 min (p < 0.00001) [40].	Tumor resection time was 7 min shorter than in the traditional treatment, at 26.2 min [41].	Tumor contouring time was 3.2 min (range: 1.6–9 min). The median operation time for non-palpable tumors was 66 min (range: 44–119 min) [29].	There are no clinical trial data available.	There are no clinical trial data available.
**Tissue Preservation**	The average volume of the resected tissue was reduced from 744 g to 46 g (P = 0.0241) [42].	The resection volume was significantly smaller than in the traditional treatment (16.2 cm³ vs. 22.8 cm³; P = 0.024). The tumor volume to specimen volume ratio was significantly higher (4.5 % vs. 3 %; P = 0.001) [41].	The average specimen volume in the NaviKnife group was 95.4 ± 73.5 cm³ , compared to 140.7 ± 100.3 cm³ in the control group (P = 0.01) [29].	The average resection volume was 94 cm³ in the MarginProbe group vs.107 cm³ in the control group [43].	There are no clinical trial data available.
**Impact on Reoperation Rate**	Many studies suggest no significant impact [26].	Significantly reduced re-excision rate (2.5 % vs. 12.5 %; p = 0.032) [44].	The proportion of residual disease in re-excision specimens was 14.3 % (NaviKnife group) and 50 % (control group) [29].	Multiple RCTs prove that MarginProbe can reduce the secondary surgery rate by more than 50 % [32].	There are no clinical trial data available.
**Advantages**	Enhance the visualization of tumor margins characterized by microcalcification or calcification clusters [26], [35].	Offers high precision in tumor localization, significantly reduces resection volume, and improves margin status assessment [41], [44]	Good integration; the probe can access the surgical cavity, and the technology is radiation-free [29].	In a setting with a low baseline re-excision rate, using MarginProbe as a surgical adjunct reduced the re-excision rate by 2 % (from 8.6 % to 6.6 %) [39]. ClearEdge system reportedly achieves better sensitivity and specificity for assessing margins than MarginProbe, and it can be tuned to probe a particular depth within the specimen, e.g. 2 mm. [45]	High sensitivity for tumor detection (100 % in clinical trials); Real-time imaging with fast acquisition times (≤5 min); Safe for use with no radiation exposure and minimal thermal effects (skin temperature ≤37°C); Utilizes endogenous contrast agents (e.g., hemoglobin) and exogenous agents (e.g., isosulfan blue) for enhanced visualization; Effective across all Fitzpatrick skin types [38].
**Limitations**	The sensitivity to non-calcified invasive lesions remains low, and there is a risk of radiation exposure [26].	IOSU can only obtain two-dimensional imaging and has a limited resolution. IOUS exhibits inherent operator dependence, and the pressure applied by the probe can induce deformation artifacts in the tissue.	Requires pre-operative implantation of markers (e.g., using the EnVisio™ Surgical Navigation system) under ultrasound or mammographic guidance [37]; susceptible to signal interference from aesthesia machine, metallic instruments, etc.	Lack tissue penetration depth; loses anatomical context due to tissue retraction causing positioning errors (2–3 mm), preventing real-time adjustment of resection margins; MarginProbe has a high false-positive rate, which may lead to unnecessary extended resection [46]; the significant differences in sensitivity and specificity reported across different studies suggest that the results are susceptible to human and intraoperative factor; the use of disposable probes substantially increases surgical costs [39], [47]	Lack of reported specificity data in clinical trials; Requires further validation in larger cohorts to establish diagnostic accuracy across diverse tumor subtypes; Potential for high equipment costs and disposable components; Depth penetration may be limited compared to some modalities (though typically up to 5 cm) [38].

### 1.4 Photoacoustic imaging for intraoperative margin assessment

Intraoperative margin assessment is paramount in BCS, aiming to achieve complete tumor resection (R0 resection with microscopically negative margins) [13] while maximizing the preservation of healthy tissue for optimal aesthetic and functional outcomes. However, current technologies face two critical limitations.

First, inadequate spatial resolution and contrast fundamentally limit the precise delineation of tumor boundaries. For instance, while intraoperative mammography can detect microcalcifications, it lacks sensitivity for non-calcified invasive foci. Ultrasound provides real-time imaging but suffers from poor contrast between tumor and normal tissue, leading to low sensitivity for ductal carcinoma in situ (DCIS). Although MRI offers high resolution, its bulkiness makes it incompatible with standard surgical workflows. Secondly, inherent diagnostic latency in pathological evaluation prevents real-time surgical feedback. FS analysis, while valuable, is time-consuming and examines only a small fraction (approximately 6.5 %) of the margin surface. Critically, it fails to detect microscopic residual disease in about 12 % of cases initially deemed "margin-negative" [12], [23], often necessitating second surgeries for re-excision.

Photoacoustic Imaging (PAI), with its superior spatial resolution, contrast, and functional insights into tumor biology (e.g., hypoxia, angiogenesis), holds promise for enhancing intraoperative margin assessment [48]. While several excellent reviews have comprehensively covered the technical principles and preclinical applications of PAI, a significant literature gap remains in its systematic application for intraoperative margin assessment during BCS. Existing articles often focus on a singular aspect, such as instrumentation or contrast agent development, but lack a unified framework that integrates the multi-scale diagnostic biomarkers PAI provides for determining tumor boundary integrity.

This review is uniquely positioned to bridge this gap. Unlike previous works, we elaborate on how PAI leverages optical absorption contrasts to generate a hierarchy of diagnostic biomarkers, ranging from subcellular morphology, metabolic activity, vascular patterns and microcalcification composition to sentinel lymph node detection, synthesized from a surgeon's perspective. This paper serves as a practical guide for clinicians, aiming to bridge the gap between technological innovation and the pressing clinical need for accurate intraoperative margin assessment.

## 2 Principles of photoacoustic imaging for intraoperative margin assessment

### 2.1 Technical principles of PAI in BCS

**Fig. 3:**
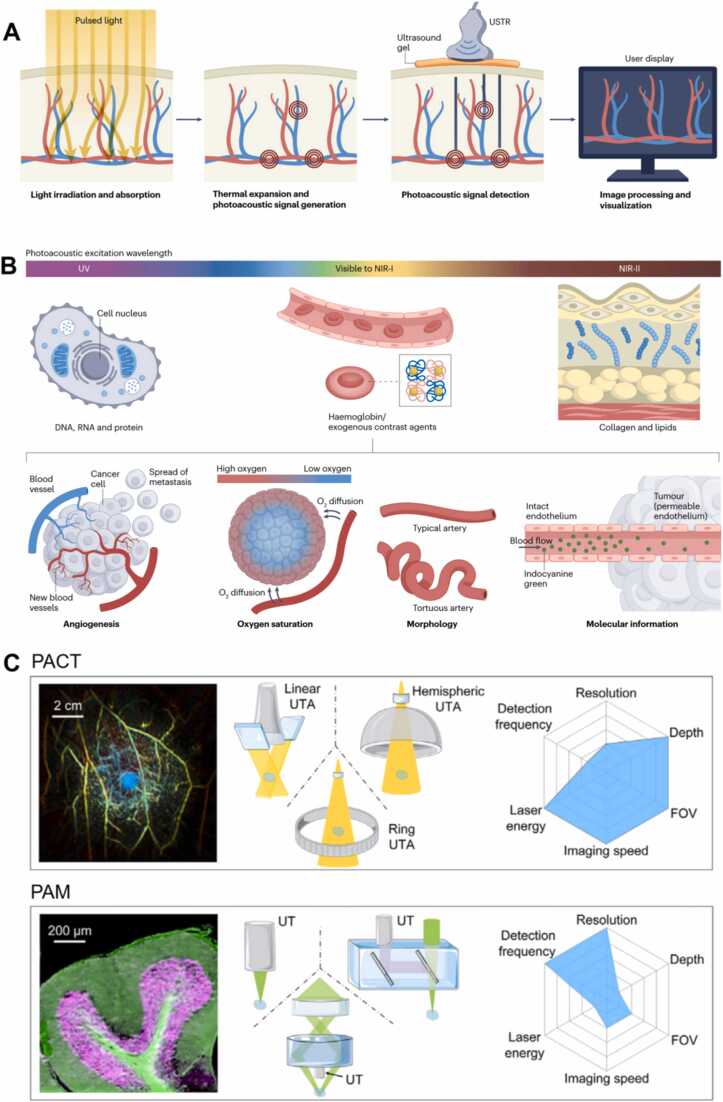
Overview of PAI. A. In PAI, short-pulsed light irradiates tissue, absorbed by endogenous chromophores (e.g., hemoglobin, melanin) or exogenous agents (e.g., indocyanine green). This absorption generates localized thermoelastic expansion, producing ultrasound waves detected by transducers. Signals are processed to reconstruct cross-sectional 2D/3D images reflecting optical absorption properties [48]. B. Spectral PAI applications: UV wavelengths (DNA/RNA/proteins), visible-NIR-I (hemoglobin/exogenous agents for angiography/oxygenation), and NIR-II (collagen/lipids). Wavelength selection enables molecular-specific contrast based on target absorption profiles [48]. C. Different configurations and imaging parameters for different optical excitations and acoustic receptions [56], [63], [64].

PAI is an emerging non-invasive, non-ionizing biomedical modality that synthesizes the high contrast of optical imaging with the deep penetration of ultrasound [48]. Its core principle involves irradiating biological tissue with short-pulsed laser light. This excites endogenous chromophores (e.g., hemoglobin, melanin, DNA, RNA, collagen, lipids) or exogenous contrast agents (such as indocyanine green, ICG, and methylene blue), causing them to absorb optical energy. The resulting localized temperature spikes induce rapid transient thermoelastic expansion within the tissue, generating broadband ultrasound signals (photoacoustic waves) [49] (Fig. 3A). These signals are detected and reconstructed to map the spatial distribution of optical absorption properties [50]. By analyzing the resulting photoacoustic contrast differences, PAI enables discrimination of cellular and histological morphology, visualization of blood flow dynamics, identification of tumor microcalcifications, and precise differentiation between malignant and normal tissue. This significantly enhances tumor-normal tissue differentiation accuracy [51]. PAI uniquely combines high spatial resolution, deep-tissue imaging capability, and functional sensitivity to physiological parameters like blood oxygen saturation (distinguishing oxyhemoglobin from deoxyhemoglobin). This facilitates real-time, high-contrast visualization of tumor margins and precise delineation of tumor-associated microvascular networks [52]. It demonstrates significant potential for intraoperative margin assessment and early breast cancer diagnosis. Compared to conventional imaging modalities, PAI provides superior tumor-to-normal tissue contrast, enables real-time surgical margin assessment. It may help reduce recurrence risks by ensuring complete tumor excision while minimizing healthy tissue removal. Additionally, targeted contrast enhancement allows for identification of sentinel lymph nodes (SLNs).

(1) Contrast of PAI

The contrast in PAI primarily relies on the optical absorption properties of tissues at specific wavelengths, as different biomolecules exhibit distinct absorption characteristics across the electromagnetic spectrum [53] (Fig. 3B).

1. Ultraviolet Band (200–300 nm): Nucleic acid bases in DNA and RNA (e.g., adenine, guanine, cytosine, thymine, uracil) exhibit firm absorption peaks near 260 nm [54], while aromatic amino acids in proteins (e.g., tryptophan, tyrosine, phenylalanine) also absorb intensely between 250 and 300 nm [55]. Leveraging these absorption properties in the UV spectrum, PAI enables label-free imaging of nuclear and cytoplasmic structures [56], achieving contrast levels comparable to hematoxylin and eosin (H&E) staining used in conventional histopathology.
2. Visible to Near-Infrared-I (NIR-I, 400–1000 nm): Hemoglobin (Hb) dominates light absorption in this range, making it ideal for vascular network imaging. This band supports high-resolution visualization of vascular morphology and enables functional assessments such as blood oxygen saturation monitoring, angiogenesis mapping, and microcirculation evaluation [57].
3. Near-Infrared-II (NIR-II, 1000–1700 nm): With deeper tissue penetration, NIR-II facilitates imaging of subsurface structures. Collagen and lipids exhibit pronounced absorption in this band, allowing PAI to differentiate breast parenchyma from adipose tissue, critical for margin assessment in BCS [57].

Exogenous agents such as indocyanine green, methylene blue, metal nanoparticles, carbon-based nanomaterials, and polymeric nanoparticles are employed to enhance contrast. These agents often surpass endogenous chromophores in absorption efficiency and stability, significantly improving signal-to-noise ratio (SNR) and imaging contrast. For example, gold nanoparticles generate intense absorption signals in the NIR band, enabling high-contrast imaging of tumors or inflammatory lesions.

(2) Classification of PAI

PAI encompasses multiple modalities tailored to diverse clinical scenarios, with imaging depth and resolution adjustable via ultrasound (US) frequency. Higher central frequencies and bandwidths of ultrasonic detectors enhance spatial resolution but reduce penetration depth [58]. Depending on the imaging modality and resolution, three primary implementations dominate current applications: Photoacoustic Computed Tomography (PACT), Photoacoustic Microscopy (PAM), and Photoacoustic Endoscopy (PAE).

PACT serves as the cornerstone for intraoperative margin assessment. It employs unfocused laser illumination and low-frequency ultrasonic transducers (1–5 MHz) to capture deep-tissue photoacoustic signals, which are reconstructed into three-dimensional oxygen saturation maps via inverse algorithms (Fig. 3C). With a penetration depth of 20–30 mm [59], PACT fully covers the standard margin range in BCS (5–20 mm). It excels in real-time monitoring of hypoxic tumor regions and has been integrated into intraoperative ultrasound systems [57]. For instance, Yang et al. [60] developed a portable PACT probe that generates tumor boundary heatmaps within 10 s during clinical trials, improving positive margin detection rates by 32 % compared to conventional ultrasound. The system also visualizes vascular infiltration patterns in deep residual lesions, offering surgeons intuitive guidance for resection.

PAM is categorized into Optical-Resolution PAM (OR-PAM) and Acoustic-Resolution PAM (AR-PAM) based on the relative sizes of optical and acoustic focal spots (Fig. 3C). PAM utilizes ultraviolet (UV) wavelengths (<300 nm), which are absorbed by endogenous DNA and RNA in cell nuclei. This enables submicron-resolution and label-free identification of nuclear atypia, including enlarged nuclei, hyperchromatic chromatin and irregular nuclear membranes [61]. It completes ex vivo margin scanning in 3 min with 93 % sensitivity, matching the accuracy of time-consuming (>30 min) FS analysis. This innovation significantly reduces intraoperative pathological waiting times [62]. However, the imaging depth is limited to less than 1 mm, necessitating complementary in vivo PACT validation for deep infiltrative margins.

PAE utilizes miniaturized catheter probes to achieve high-resolution imaging (3 μm), even detecting microcalcifications, a critical diagnostic marker for breast cancer. However, due to slow mechanical scanning speeds, PAE is unsuitable for open BCS and is currently restricted to luminal organ diagnostics (e.g., vasculature, gastrointestinal tract).

In conclusion, PACT provides micrometer-scale resolution and offers deeper penetration capability. This enables imaging of relatively large tissue volumes. Consequently, PACT is suitable for in vivo assessment of the surgical cavity or the entire breast region. For example, it can scan the cavity wall after tumor resection to detect residual lesions. In contrast, PAM delivers exceptionally high spatial resolution, achievable at the nanometer scale. This permits imaging at the cellular and subcellular levels and makes PAM particularly well-suited for detailed analysis of resected ex vivo specimens. The three techniques, PACT, PAM, and PAE, exhibit complementary advantages in imaging depth, resolution, and clinical applicability. Their synergistic integration, driven by unique photoacoustic mechanisms, underscores the transformative potential of PAI in intraoperative margin assessment for BCS.

### 2.2 Integration of cellular morphology and tissue metabolism for tumor boundary delineation

Breast cancer subtypes—such as invasive ductal carcinoma (IDC), invasive lobular carcinoma (ILC), and papillary carcinoma—exhibit distinct histomorphological features, posing challenges for intraoperative boundary delineation. IDC is characterized by tumor cells invading periductal stroma to form nests or solid sheets and accounts for 70–80 % of breast cancers. These tumor cells typically display marked nuclear atypia, including enlarged nuclei, hyperchromasia, and an elevated nuclear-to-cytoplasmic ratio. In contrast, ILC displays single-file infiltration along glandular septa, often leading to diagnostic oversight, while papillary carcinoma features papillary growth patterns [62]. Traditional intraoperative FS analysis relies on the expertise of pathologists. While it can provide an initial assessment based on histomorphology, it may be insufficient for definitive subtyping of breast cancer in cases with ambiguous morphological features. A conclusive diagnosis often requires postoperative paraffin-embedded sectioning and IHC staining, which delivers results days later. However, PAI enables label-free visualization of tumor morphology and metabolism by resolving optical absorption properties at cellular and tissue levels, offering a novel approach for real-time intraoperative diagnosis.

(1) Cellular and Tissue Morphological Imaging

Through tightening the acoustic or light focusing, PAI enables multi-scale resolution from microvascular networks to subcellular structures, achieving label-free identification of nuclear isoforms in breast cancer cells.

**Fig. 4:**
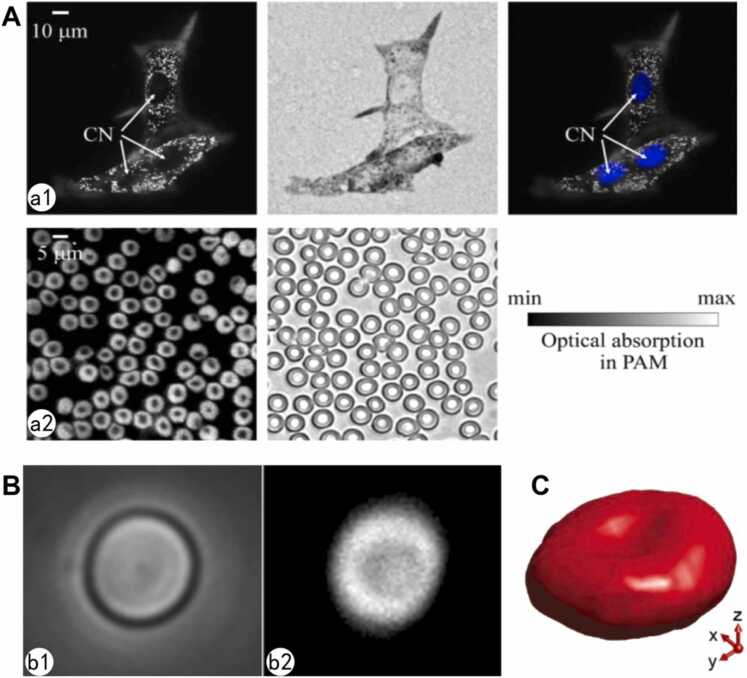
Photoacoustic image of ex vivo melanoma cells and RBCs. A. Ex vivo images of cells. (a1) Melanoma cells. From left to right: photoacoustic microscopy (PAM) image, optical microscopy (OM) image (0.55 NA), and a composite of the PAM image and the fluorescence OM image of the stained nuclei (blue). In the PAM images the strong signals come mainly from melanin, and the white dots are melanosomes. CN: cell nucleus. (a2) PAM and OM (1.0 NA) images of red blood cells. The strong signals in the PAM image come mainly from hemoglobin [66]. B. A red blood cell measured using the 1200 MHz transducer. (b1) Optical and (b2) Photoacoustic image of a single RBC fixed on the glass substrate. The width of the image is 15 μm [67]. C. 3D visualization of the RBC [68].

In 2008, Lihong V. Wang's team invented OR-PAM by utilizing laser focal scanning technology to enhance the spatial resolution to 5 μm [65], enabling the first visualization of capillary networks in live small animals. Although this resolution has not yet reached the single-cell level, it demonstrated the potential of PAI for morphological analysis. In 2010, the team further compressed the excitation laser focus using a high-numerical-aperture objective lens (NA = 1.23), achieving a 220 nm lateral resolution, which marked PAI's entry into cellular imaging [66]. Leveraging this advancement, the spatial distribution of melanin granules within melanoma cells and the biconcave disk morphology of red blood cells (RBCs) were resolved under label-free conditions (Fig. 4A). Melanin exhibited high signal intensity under 410 nm excitation, while RBCs displayed edge enhancement at 560 nm due to hemoglobin's absorption characteristics.

Strohm et al. [67] employed a 1200 MHz central-frequency ultrasound transducer, a 0.25-NA objective, and 532 nm laser excitation to obtain high-resolution photoacoustic images of individual RBCs (Figure. 4b1). Compared to bright-field optical microscopy (Figure. 4b2), the photoacoustic images not only preserved key morphological features (e.g., the central concavity of RBCs) but also exhibited superior contrast. Dong et al. [68] introduced an optical microring resonator for photoacoustic signal detection, achieving high-resolution photoacoustic microscopy (PAM) with 0.7 μm lateral and 2.1 μm axial resolutions. The system's ultra-wide detection bandwidth (280 MHz) and high sensitivity enabled the 3D reconstruction of RBCs (Fig. 4C), confirming their biconcave structure.

The high-resolution imaging of RBCs and melanoma cells, as demonstrated in these pioneering studies, serves as a critical proof-of-concept for PAI's capability. It verifies PAI's intrinsic sensitivity to endogenous biomolecules like hemoglobin and its capacity for label-free nuclear delineation based on optical absorption contrasts. This foundational capability is directly translatable to BCS margin assessment. Characterized by enlargement, hyperchromasia, and an increased nuclear-to-cytoplasmic ratio in IDC, the nuclear atypia falls within the resolution range of advanced PAM systems. Consequently, these methodologies in foundational studies provide the technological groundwork for PAI to perform rapid, label-free intraoperative analysis at the cellular level. This addresses a key limitation of FS by enabling wide-field detection of microscopic residual disease based on cell morphology, thereby directly contributing to the goal of achieving negative margins in BCS.

(2) Tissue Metabolic Imaging

The dynamic metabolic activity of breast cancer, including accelerated protein synthesis and enhanced oxidative stress, can be noninvasively captured through photoacoustic spectral signatures.

Rodrigues et al. [69] developed a miniaturized photoacoustic probe that integrates a sterile operative chamber with a multispectral laser module (wavelength range: 260–900 nm), enabling in vivo metabolic tracking in orthotopic breast cancer xenograft models. Following MCF-7 cell injection into athymic nude mice, photoacoustic spectra were recorded at 5-day intervals over 20 days. Machine learning-aided analysis revealed tumor-stage-specific spectral variations: sensitivities of 95 %, 100 %, 92.5 %, and 85 % were achieved at days 5 (tumor initiation), 10, 15, and 20 (necrosis phase), respectively. Notably, the photoacoustic signal from tryptophan residue at 281 nm increased with higher Ki-67 expression, a proliferation marker confirmed by histopathology. Furthermore, spectral broadening in advanced tumors indicated necrotic expansion. This link was verified by the correspondence between H&E-confirmed necrotic regions at day 20 and increased low-frequency photoacoustic components, demonstrating the method's predictive capability for tumor progression [70]. These findings elucidate metabolic heterogeneity in breast cancer and, more importantly, establish a novel intraoperative strategy. This approach integrates quantitative spectral modeling with intraoperative probe scanning of resection margins to localize metabolically active residual lesions, thereby reducing reliance on subjective pathological expertise.

### 2.3 Microcirculation and tumor vasculature imaging for margin assessment

Breast cancer progression and metastasis are closely linked to hypoxic states and vascular heterogeneity within the tumor microenvironment. Rapid tumor proliferation increases oxygen consumption, driving angiogenesis. However, tumor vasculature exhibits structural and functional abnormalities (e.g., incomplete vessel walls, aberrant branching) that fail to meet the metabolic demands of proliferating cells, exacerbating hypoxia and forming a vicious cycle that promotes metastasis and drug resistance [71]. PAI noninvasively quantifies oxygen saturation (sO₂), vascular topology, and vascular branchpoint (VBP) density by resolving hemoglobin spectral properties, providing multi-dimensional functional data for intraoperative tumor boundary delineation. This section elaborates on PAI's technical strengths and clinical value through oxygen metabolism, vascular distribution, and branching patterns.

(1) Oxygen Saturation

**Fig. 5:**
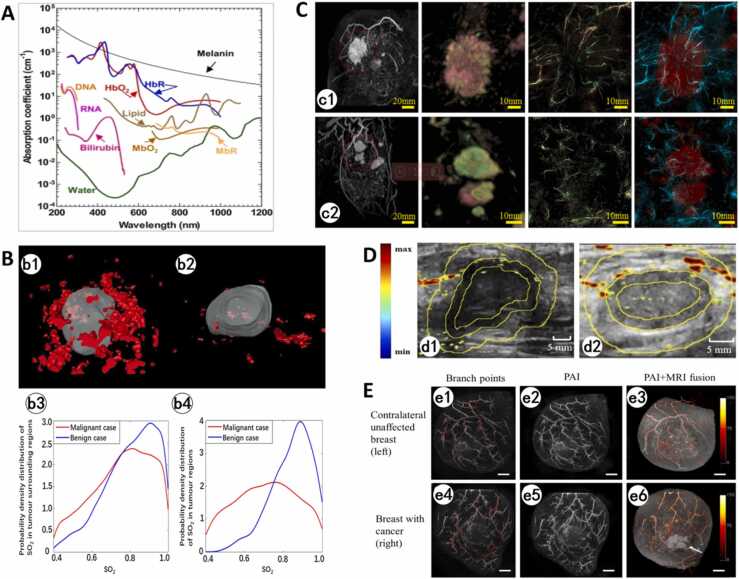
A. Absorption coefficients of different endogenous chromophores [83]. B. 3D reconstruction vascular network maps of (b1) invasive breast cancer and (b2) breast fibroadenoma. The sO_2_ density distribution in the (b3) tumor regions and (b4) tumor surrounding regions of these two tumors [60]. C. Examples of peritumoral images from two cases. Original MR images (left column) show lesions marked by red circles, followed by enlarged MR images aligned to PA dimensions (second column). The third and fourth columns display original PA images and fused PA (cyan)/MR (red) composites. Subcutaneous vessel PA signals (>4 mm depth) were excluded in all images. Case (c1): Tumor-related blood vessels seem to converge from the normal breast tissue toward the center of the tumor, becoming drastically narrower at the tumor edge and nearly vanishing near the center. Case (c2): Tumor-related blood vessels seem to converge toward the tumor center through several distinct paths or bundles [79]. D. The dual-modal PMEA/US imaging of patients. (d1) A malignant breast tumor. The central region exhibited diminished PA signals, while the surrounding and peripheral regions displayed abundant signal intensity. (d2) A benign breast tumor. Abundant PA signals were detected in all of the regions [80]. E. (e1, e4) Number of branching points in a breast with cancer and the contralateral breast with no detectable cancer. Points counted on the PAI image are shown in red. (e2, e5) PAI image. (e3, e6) Color PAI image with deformed MRI. The arrow indicates the site of the cancer. The color bar represents the PAI signal amplitude [82].

Among all endogenous chromophores, hemoglobin is a dominant absorber at wavelengths below 1000 nm. By leveraging the distinct absorption spectra of oxygenated (HbO₂) and deoxygenated hemoglobin (HbR), PAI enables imaging of vascular architecture, hemoglobin oxygen saturation (sO₂), blood flow velocity, and oxygen metabolic rates [72], [73], [74]. Within the 650–900 nm range, HbR and HbO₂ exhibit absorption coefficients at least one order of magnitude higher than other chromophores (e.g., lipids, water) at physiological concentrations [75], [76] (Fig. 5A). Although melanin shows higher absorption in this range, its localized abundance in the skin or retina excludes it from oxygen quantification. The selective absorption of HbO₂ and HbR allows high-contrast visualization of perfused vasculature in photoacoustic tomography (PAT) [77]. Studies demonstrate that functional PAT (fPAT) generates absolute total hemoglobin concentration (HbT) and oxygen saturation (StO₂%) maps in breast cancer. In six pathologically confirmed cases, fPAT achieved a 100 % tumor detection rate, whereas MRI identified five cases and misclassified one small tumor as near-complete regression. The mean HbT and StO₂% in suspicious lesions were 61.6 ± 18.9 μM/L and 67.5 % ± 5.2 %, respectively, compared to 25.6 ± 7.4 μM/L and 65.2 % ± 3.8 % in adjacent normal tissue. The research demonstrated fPAT is a potential method for tumor margin detection and characterization [78].

Yang et al. [60] developed a novel 3D photoacoustic/ultrasound (PA/US) dual-modality functional imaging system and validated it in a quantitative study involving 24 Asian female patients with breast tumors. The system integrates a 192-element linear ultrasound probe (element size: 0.2 mm, central frequency: 5.8 MHz) with a tunable pulsed laser (700–850 nm, repetition rate: 10 Hz). Using dual-wavelength excitation (750 nm and 830 nm), the system synchronously acquires PA and US signals at five frames per second via time-division multiplexing. A motorized stage scans at 1 mm/s, collecting data over a 4 cm range with 0.2 mm step resolution, completing full-field imaging in 40 s. Results demonstrated that T1-stage invasive breast cancer tumors exhibited a 7.7 % reduction in mean sO₂ at the core compared to benign tumors (*p* = 0.016), and a 3.9 % decrease compared to normal breast tissue (*p* = 0.010). Additionally, peritumoral regions of malignancies showed 4.9 % lower sO₂ than benign lesions (*p* = 0.009). ROC analysis revealed excellent diagnostic performance in discriminating invasive breast cancer (IBC) from benign tumors, achieving 100 % sensitivity and 62.5 % specificity (AUC=0.88). The optimal sO₂ threshold was determined to be < 73.2 % (Fig. 5B). This metabolic parameter effectively distinguished microinvasive foci from fibroadenomas, providing molecular metabolic insights beyond conventional ultrasound.

In a prospective multicenter study by Huang et al. [52], 120 American patients with breast lesions underwent dual-wavelength PAI to map sO₂ distributions within lesions and surrounding 2 cm tissues, compared to grayscale ultrasound (GSUS) under blinded conditions. Malignant lesions showed a mean sO₂ of 71.30 %, 12.51 % lower than benign lesions (83.81 %, P < 0.01). Within 5 mm of malignant margins, sO₂ (73.8 %) remained significantly lower than benign margins (79.4 %, P < 0.01), reflecting the metabolic invasion pattern. PA/US achieved superior diagnostic performance (AUC=0.89) versus GSUS (AUC=0.70). At a sO₂ cutoff of 78.85 %, PA/US balanced sensitivity (89.58 %) and specificity (86.11 %), reducing false-positive rates by 30.75 % and false-negative rates by 4.16 % compared to GSUS. This advantage was pronounced in dense breasts (ACR density C/D), where PA/US improved microcalcification detection with hypoxic cores by 42 %, offering critical functional guidance for intraoperative subclinical lesion localization.

In summary, intraoperative PAI can dynamically monitor sO₂ gradients at resection margins, precisely identifying hypoxic transition zones (e.g., regions with steep sO₂ declines) to guide optimal excision boundaries.

(2) Vascular Distribution

Neovascularization in malignant breast tumors exhibits distinct spatial patterns, which PAI can reveal through high-resolution vascular imaging. This offers morphological insights for malignancy differentiation and invasive margin localization.

Toi et al. [79] developed a hemispherical detector array (HDA) system, comprising 512 elements with a central frequency of 2 MHz. Under dual-wavelength excitation at 755 nm and 795 nm with a laser energy of 200 mJ per pulse, HDA system achieved multimodal vascular fusion imaging by integrating PAI with MRI, doubling the spatial resolution of conventional MRI. Their findings showed that 61 % of invasive breast cancer cases exhibited centripetal vascular orientation. Specifically, tumor-associated vessels converged toward the tumor center, abruptly narrowed or truncated at the boundaries and were nearly absent in the core region (Fig. 5C). In contrast, only 35 % of ductal carcinoma in situ cases displayed centripetal features, suggesting vascular morphology as a potential marker of invasiveness.

Rui Zhang et al. [80] conducted consecutive imaging on breast lesions and surrounding tissues in 40 patients, performing quantitative and semiquantitative analyses of vascular distribution via PAI. The study revealed greater vascular heterogeneity in malignant tumors, with a higher spatial density of PA signals in peripheral and marginal regions compared to central areas (Fig. 5D). Although benign tumors also showed elevated vascular signals at the edges, the disparity between central and peripheral regions was less pronounced than in malignancies. This reflects the irregular vascular architecture of malignant tumors, likely attributable to rapid growth overwhelming the central blood supply, thereby reducing PA signal intensity in core regions [81].

These findings suggest a "peripheral enrichment-central sparsity" pattern in vascular distribution, which is potentially linked to hyperactive angiogenesis at the invasive front and ischemic necrosis in tumor core. This characteristic distribution enables intraoperative PAI to generate real-time vascular density heatmaps, rapidly identifying abnormal gradients, such as abrupt signal spikes, vessel truncation, or rapid narrowing. Detection of these features prompts surgeons to perform an extended excision, followed by rescanning for verification.

(3) Vascular Branch Points

Vascular branch point (VBP) density reflects the complexity of vascular networks and correlates strongly with breast cancer proliferation and metastatic potential. PAI quantifies VBP distribution through three-dimensional vascular reconstruction, offering a novel intraoperative biomarker for assessing tumor margins.

Yagam et al. [82] utilized a hemispherical detector array-based 3D PAI system (PAI-03) to characterize VBPs in the superficial subcutaneous vasculature of the breast. The PAI-03 system features a 512-element hemispherical detector array (HDA), each element measuring 3 mm in diameter and operating at a central frequency of 2 MHz. It achieves submillimeter resolution and depths exceeding 20 mm in breast tissue. Dual-wavelength excitation at 755 nm and 795 nm enables simultaneous imaging of HbO₂ and HbR absorption coefficients. Based on these coefficients, three-dimensional breast profiles are reconstructed using universal back-projection algorithms, which account for variation in tissue-specific acoustic impedance and photonic flux. In 22 patients with unilateral breast cancer, affected breasts exhibited significantly higher mean VBP counts at 7 mm subcutaneous depth compared to contralateral healthy breasts (P < 0.01). Elevated VBP ratios (cancer-to-healthy breast) were associated with high histological grade (*p* = 0.03), estrogen receptor negativity (P < 0.01), and highly proliferative tumors (P < 0.01), suggesting increased superficial VBP density as a potential biomarker for primary breast cancer (Fig. 5E).

PAI delineates vascular architecture and sO₂ by resolving HbO₂-HbR absorption differences, revealing significantly lower sO₂ in malignant regions than in benign or normal tissues. Malignant tumors exhibit centripetal vascular orientation, characterized by abrupt narrowing or truncation at boundaries and dense peripheral vascular signals. Furthermore, VBP spatial density is markedly higher at tumor-invasive fronts, correlating with enhanced proliferative and invasive phenotypes. PAI provides intraoperative margin guidance by integrating real-time VBP mapping and sO₂ gradients, enabling surgeons to define and excise high-risk zones precisely. This approach minimizes residual cancer cells while preserving healthy tissue, significantly improving the safety and efficacy of BCS.

### 2.4 Microcalcification detection

Breast microcalcifications (diameter <1 mm) are critical markers for clinically occult breast cancer, particularly T0-stage lesions, with approximately 50 % of non-palpable tumors detected through mammography. However, conventional mammography is limited by radiation exposure, patient discomfort from breast compression, and an inability to differentiate pathologic nature of microcalcification, such as hydroxyapatite (HA) and calcium oxalate (CA). With its submillimeter spatial resolution and endogenous molecular spectral discrimination, PAI offers a revolutionary approach for real-time intraoperative microcalcification localization and pathological grading.

**Fig. 6:**
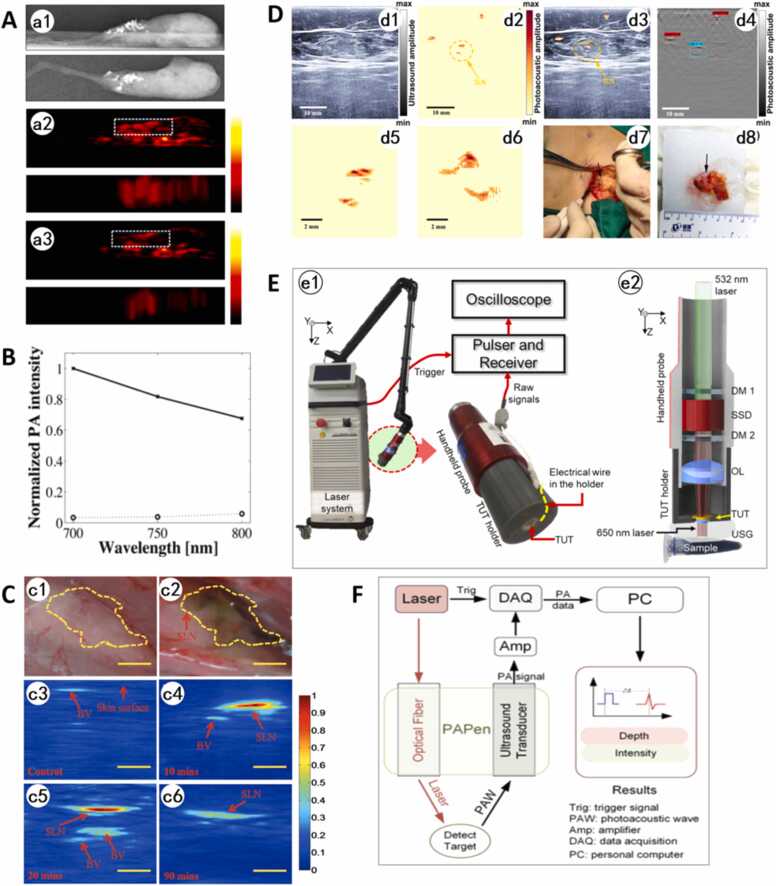
A. Ex vivo breast tissue microcalcification imaging. Specimen X-ray (a1) and photoacoustic images at 700 nm (a2) and 800 nm (a3) were acquired under consistent imaging conditions. PA signal locations corresponded to microcalcifications identified on X-ray, finding that the 800 nm image demonstrated reduced signal intensity in microcalcification regions compared to 700 nm imaging [86]. B. Normalized PA intensities measured in the in vitro experiment with the raw materials. Solid and dotted lines indicate the measurements from hydroxyapatite and calcium oxalate, respectively [84]. C. In vivo visual detection of SLN (c1) before and ((c2) after the injection of N4 NPs. In vivo photoacoustic imaging of SLN (c3) before and (c4) after the injection of N4 NPs for 10 min, (c5) 20 min, and (c6) 90 min. Excitation wavelength is 800 nm. BV, blood vessels. Scale bars: 1 mm [93]. D. Representative images of in vivo PA/US imaging of SLNs for a woman with breast cancer. (d1) US image. (d2) PA image (SLN circled). (d3) Co-registered PA/US image (SLN circled). (d4) SLN is automatically detected by the convolutional neural network (CNN) network in the PA image (scale bars: 10 mm). (d5-d6) Validation of the detected SLNs by removing the SLN and ex vivo PA imaging: (d5) In vivo PA image of the SLN of Patient 1, and (d6) Ex vivo PA image of the resected SLN in an agarose phantom. (d7) A photo of the stained LN (arrow) during surgery. The incision was guided by the PA/US imaging system. (d8) A photo of the ex vivo specimen [94]. E. Schematic of the handheld photoacoustic finder system. (e1) The portable solid-state dye laser system with a handheld probe. (e2) Detailed schematic of the handheld probe. TUT, transparent ultrasound transducer; DM, dichroic mirror; SSD, solid-state dye; OL, objective lens; and USG, ultrasound gel [95]. F. The overview of the PAPen system [97].

Kang et al. [84], [85], [86] systematically validated PAI's unique advantages in microcalcification detection and composition analysis through multi-wavelength spectral strategies. Their initial study employed dual-wavelength excitation (700/800 nm), proposing the PAI ratio (PA700/PA800 signal intensity ratio) as a microcalcification-specific marker. Ex vivo experiments demonstrated a median PAI ratio of 2.46 in microcalcifications versus 1.11 in non-calcified tissues (*p* = 0.001), achieving 90.91 % sensitivity and 80.0 % specificity for submillimeter calcifications (0.3–1 mm) [81]. In 2015, the team expanded to tri-wavelength imaging (700/750/800 nm), revealing wavelength-dependent PA signal attenuation in microcalcifications and minimal signal fluctuation in adjacent adipose/glandular tissues (Fig. 6A). PAI exhibited 96 % spatial concordance with mammographic calcification localization, confirming its reliability across morphological and spectral dimensions [86].

Further studies focused on pathological differentiation of microcalcification composition: HA correlates with malignant potential, and CA predominates in benign cases [87], [88]. Kang et al. [84] reported distinct absorption coefficients for HA (1.84 at 700 nm, 1.73 at 800 nm) and CA (1.39 at 700 nm, 1.35 at 800 nm). The HA signals at 700–800 nm wavelength band were attenuated by 32 %, while CA only attenuated by 2 % (P < 0.01, Fig. 6B). This difference is attributed to the characteristic absorption peaks in the NIR region of the phosphate groups in HA crystals, enabling its PAI ratio to differentiate between benign and malignant calcifications effectively.

Intraoperative PAI generates real-time calcification composition heatmaps, precisely localizing lesions and predicting malignant risk. This molecular imaging-guided paradigm facilitates targeted excision of non-palpable microcalcifications and personalized therapeutic strategies.

### 2.5 Sentinel lymph node detection

The sentinel lymph node (SLN), the primary site of lymphatic metastasis, reflects axillary lymph node (LN) involvement. BCS typically combines tumor resection with sentinel lymph node biopsy (SLNB), the standard approach for early-stage breast cancer (clinically node-negative, cN0). The NSABP-B32 trial (2010) validated the clinical utility of omitting axillary lymph node dissection (ALND) in SLNB-negative cases [89], establishing SLNB as the frontline strategy for regional LN assessment. While BCS preserves breast aesthetics via localized tumor excision and radiotherapy, SLNB minimizes surgical trauma through minimally invasive LN evaluation. They embody the "minimum effective therapy" principle, balancing oncological safety with reduced morbidity. International guidelines recommend combining blue dye and radiotracer methods for SLN localization [90], [91]. As a non-invasive biomedical imaging modality, PAI demonstrates significant potential for SLN detection by precisely visualizing LN structure, functional status, and metastatic involvement, which provides an essential tool for breast cancer staging and the removal of malignant LN.

In 2015, Garcia-Uribe et al. [92] modified a clinical ultrasound system (Philips iU22) by integrating a 667 nm laser, which corresponds to the absorption peak of methylene blue, and a dedicated ultrasound probe. This integration successfully achieved real-time photoacoustic tomography/ultrasound (PAT/US) fusion imaging at a frame rate of five frames per second. Methylene blue exhibits peak absorption at 665 nm and negligible absorption above 750 nm. By exploiting spectral differences between methylene blue and hemoglobin, the system alternately excites 1064 nm and 650 nm wavelengths at 10 Hz, generating PAT images and highlighting methylene blue signals via image subtraction. This marked the first integration of PAT with clinical ultrasound for real-time, non-invasive SLN detection. However, due to secondary LN staining, methylene blue's small particle size and non-targeted diffusion risk false negatives.

Cai et al. [93] developed conjugated oligomer N4-based nanoparticles (NPs) via DSPE-PEG2000 encapsulation, enabling synergistic SLN photoacoustic imaging and photothermal therapy. These NPs exhibit strong near-infrared absorption peaking at 710 nm and generate photoacoustic signals through nonradiative relaxation. After injection, SLN signals achieve peak within 10 min and retain 45 % intensity for 90 min, outperforming gold nanomaterials in rapid metabolic clearance. The experiments confirmed that the NPs have a photothermal conversion efficiency of 30 %, and elevate solution temperatures by 40 ℃ under 808 nm laser irradiation, combining excellent photostability and biocompatibility. Through surface modification of cRGD-targeting peptides, NPs specifically recognized breast cancer cells (MDA-MB-231) with high expression of integrin αvβ3. They achieved more than 60 % lethality after 8 min of 0.8 W/cm² laser irradiation without significant toxicity to normal cells (NIH-3T3). This work is the first application of conjugated oligomer nanosystems for SLN diagnostic and therapeutic integration, which provides a novel multifunctional platform for early detection and precise intervention of breast cancer metastasis (Fig. 6C).

Gu et al. [94] designed a nonradioactive dual-modality PA/US system incorporating carbon nanoparticles (CNPs) as a contrast agent for precise localization of SLN. Owing to their large average diameter of 150 nm, CNPs were selectively retained within the lymphatic system. This retention generated a unique "double line" image feature under single-wavelength (1064 nm) PAI, thereby enhancing SLN differentiation from the surrounding vascular backgrounds. In a clinical trial involving 11 breast cancer patients, the system localized 23 SLNs with 100 % specificity and 52.6 % sensitivity (Fig. 6D), where false negatives were attributed to hemorrhage, tattoo pigment interference, or lymph nodes deeper than 4.5 cm. The reliability of in vivo imaging features was confirmed by in vitro validation of resected SLNs by PAI, which showed a consistent correlation between nanoparticle staining density and photoacoustic signal intensity. A primary advantage of this technique is its inherent safety profile, as it eliminates radiological exposure, benefiting pregnant patients and radiation-controlled settings. Additionally, the highly selective retention of CNPs reduces the risk of intraoperative confusion between lymphatics and blood vessels. Future advancements aim to improve deep lymph node detection through wide-aperture ultrasound arrays and dual-wavelength spectral decomposition algorithms.

Another PA sensing method for SLN detection is called photoacoustic finder (PAF), or photoacoustic pen, to localize SLN with a handheld non-imaging probe with easier operation procedure and much lower system cost. In 2021, Chulhong Kim et al. [95] developed a handheld, radiation-free PAF integrating a solid-state dye laser with a PMN-PT crystal-based transparent ultrasound transducer (TUT). The TUT provides 72 % optical transparency at 650 nm, enabling coaxial optical-acoustic beam alignment. The laser converts 532 nm light to 650 nm, targeting methylene blue (MB) and melanoma absorption peaks (Fig. 6E). This addresses limitations of radioactive tracers and shallow-penetration (<1.5 cm) fluorescence imaging. PAF detected 30 mM MB signals beneath 22 mm of chicken tissue (SNR=12 dB, 1/e depth: 0.92 cm). In rats, it located MB-tagged SLNs achieving SNR= 40 dB without tissue cover and SNR= 18 dB under 18 mm tissue. Ex vivo validation confirmed that the PA signal intensity from MB-labeled lymph nodes was 9 times stronger than from normal nodes. Furthermore, in a mouse melanoma model, the PAF detected subcutaneously injected melanoma without exogenous contrast agents at an 18 mm depth (SNR=15 dB). With its compact size (38.5 ×38.5 ×140 mm) and laser fluence compliant with safety standards (15 mJ/cm²), the PAF presented a novel tool with high clinical translation potential for non-invasive SLN biopsy and tumor detection.

In a subsequent study in 2024 [96], the team conducted a prospective trial involving 121 breast cancer patients with 375 excised lymph nodes (including 220 SLNs). The results showed that the PAF achieved an SLN detection rate of 87 % (191/220), matching the gamma probe (85 %) and surpassing visual inspection (73 %). Crucially, in the 14 patients with histologically confirmed metastatic SLNs, both the PAF and gamma probe achieved a 100 % detection rate, while visual inspection reached only 79 % (11/14). Non-inferiority analysis confirmed PAF's parity with gamma probe (*p* = 0.015) and superiority over visual inspection (*p* < 0.001).

A key finding was that the PAF successfully identified 41 % (88/214) of SLNs missed by visual inspection, faintly stained nodes visible only upon brightening the corresponding photographs, highlighting its superior sensitivity to blue dye. This closed-loop research, which integrated technological innovation with clinical validation, demonstrates that the PAF system can use blue dye alone to achieve SLN localization equivalent to radioisotope-based methods. It offers a practical solution for standardizing radiation-free SLN biopsy.

Gao et al. [97] developed an innovative handheld photoacoustic pen (PAPen) system that integrates an annular ultrasound transducer (2.25 MHz center frequency, 80 % bandwidth) with a laser-delivery optical fiber within a 19-cm cylindrical probe (Fig. 6F). This device quantitatively localizes SLNs by detecting photoacoustic signals from Indocyanine Green (ICG) contrast agents under 780 nm laser excitation. The depth calculation is based on the principle *h* = *c / f* × *n*, where *h* = depth, *c* = the sound speed of the medium, *f* = the sampling ratio of the data acquisition equipment, and *n* = PA signal index. The PAPen device overcomes critical limitations of conventional SLN detection methods by offering a 50-mm penetration depth beyond fluorescence imaging constraints. It can enable real-time 10-Hz signal updates, eliminate ionizing radiation risks, and concurrently quantify depth/signal intensity rather than approximating location. Validation studies demonstrated the device’s capability by successfully detecting ICG through 5-cm thick chicken breast tissue in phantoms. Furthermore, in vivo murine experiments confirmed accurate measurement of SLN depth at 9-mm, which correlated closely with photoacoustic tomography. Although signal aliasing between ICG and blood remains a challenge, employing multispectral excitation is a promising strategy to enhance specificity. This non-invasive, depth-quantifiable technology is thus positioned as a promising intraoperative tool for precision SLN biopsy.

Through multimodal integration and contrast agent innovation, PAI is overcoming the invasiveness and specificity limitations of traditional SLN detection, offering a real-time, non-invasive tool for precision breast cancer management. Advancing material science and imaging technologies will accelerate clinical translation, further realizing the "Minimum Effective Therapy" paradigm Table 2.

**Table 2:** Key biomarker characteristics of photoacoustic imaging for margin assessment in breast-conserving surgery and their clinical implications.

**Features**	**Biomarker Correlation**	**Description**
Cell morphology	Breast cancer pathological subtypes exhibit distinct histological morphological features (e.g., invasive ductal carcinoma, invasive lobular carcinoma, papillary carcinoma)	PAI identifies malignant characteristics including nuclear atypia and increased nuclear-cytoplasmic ratio, enabling subtype-specific diagnosis.
Organic metabolism	Dynamic metabolic activity correlates with tumor invasiveness (accelerated protein synthesis, enhanced oxidative stress)	Intraoperative real-time tumor margin delineation is achieved through photoacoustic metabolic tracking, guiding precision resection.
Oxygen saturation	Rapidly proliferating tumor cells often experience insufficient nutrient supply, leading to hypoxic conditions within tumor regions.	PAI reveals lower blood oxygen saturation (sO₂) in tumor regions compared to the surrounding peri-tumoral tissue.
Vascular Distribution	Intratumoral vascular heterogeneity is characterized by features such as vascular regression in the central region due to hypoxia or necrosis, and aberrant angiogenesis or architectural distortion at the periphery triggered by the tumor's invasive growth.	PAI can detect reduced vascular density in the tumor center, a sudden increase in vascular signal density at the periphery, as well as abrupt vascular termination or sharp narrowing of blood vessels.
Vascular Branch Points（VBPs）	The density of VBPs reflects the complexity of the vascular network and is closely associated with the proliferative activity and metastatic potential of breast cancer.	A higher density of VBPs detected by PAI in the superficial subcutaneous layer of the breast may serve as a potential biomarker for primary breast cancer.
Microcalcification	Microcalcifications (diameter <1 mm) serve as a critical marker for clinically occult breast cancer and carcinoma in situ.	PAI can precisely localize microcalcification foci, delineate tumor margins, and the PAI ratio may effectively distinguish between benign and malignant calcifications.
Sentinel Lymph Node (SLN)	SLN is the first station in the lymphatic metastatic pathway of breast cancer. If the SLN shows no metastasis, complete axillary lymph node dissection can be avoided, significantly reducing the risk of postoperative upper limb lymphedema; if positive, further axillary lymph node dissection or radiotherapy is required.	PAI enables precise visualization of lymph node structure, functional status, and metastatic involvement, thereby avoiding the trauma associated with biopsy procedures.

## 3 Application of intraoperative margin evaluation in BCS

### 3.1 Ex vivo assessment

**Fig. 7:**
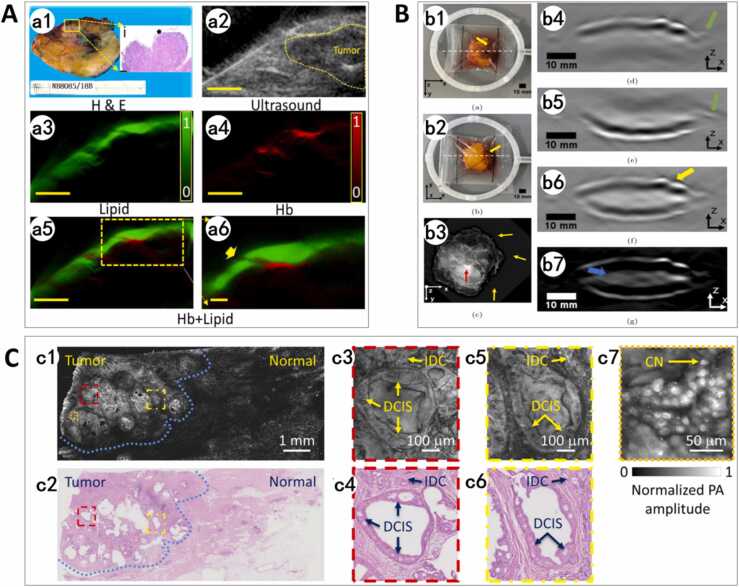
Ex vivo photoacoustic imaging [98], [99], [100]. A. US-OT imaging of malignant tumor with a positive resection margin. (a1) Gross pathology of a malignant tumor with positive margins. Black arrow indicates area of margin involvement. Scale bar, 500 μm. (a2) US image of the corresponding tumor in (a1). (a3-4) Distribution maps of lipid and Hb in the corresponding slice of the tumor. (a5) Merged and (a6) zoomed in images of (a3-4) shows interruption of lipid layers by Hb signals (indicated by yellow arrow) as a characteristic of positive resection margins. Scale bars, 5 mm [98]. B. Lumpectomy specimen imaging results. (b1-2) Photographs show surgical sutures (yellow arrows). (b3) 2D X-ray identifies the tumor region (dashed circle) and radioactive seed (red arrow). (b4-5) Raw PA images from handheld probe. Green arrows: Fiducial markers. White dashed lines: imaging planes. (b6) Registered PA overlay of (b4-5). Yellow arrows: Surgical sutures signals. (b7) Zero-forced PA image. Blue arrow: ringing artifacts. [99] C. Imaging of a breast tumor. (c1) UV-PAM image of the fixed, unprocessed breast tumor. (c2) H&E-stained histologic image of the same area shown in (c1). The blue dashed lines in (c1) and (c2) outline the interface between the normal and tumor regions. (c3-6) Zoomed-in UV-PAM and H&E-stained images of the red or yellow dashed areas in (c1) and (c2). IDC, invasive ductal carcinoma; DCIS, ductal carcinoma in situ. (c7) Zoomed-in UV-PAM image of the orange dashed region in (c1). CN, cell nuclei. [100].

Balasundaram et al. [98] investigated the application of ultrasound-guided photoacoustic tomography (US-OT) for evaluating surgical margins in breast cancer. Utilizing a 256-element curved-array US-OT probe with a central frequency of 5 MHz, spatial resolution of 150 μm, and penetration depth of 7 mm, the system integrated ultrasound with multispectral optoacoustic (MSOT) imaging. Fourteen fresh ex vivo malignant specimens (including invasive ductal carcinoma, lobular carcinoma, and ductal carcinoma in situ) were scanned using a multiwavelength (700–1100 nm) 2D PAI system. Spectral unmixing techniques were employed to map spatial distributions of biomarkers such as hemoglobin (Hb) and lipids. Results demonstrated 11 negative margins characterized by continuous, homogeneous lipid bands surrounding tumors, indicating intact adipose layers without tumor infiltration. Tumor margins exhibited annular hypoxic regions with elevated deoxygenated hemoglobin, reflecting hypoxia-driven angiogenesis. Three positive margins showed lipid layer invasion or truncation by Hb-rich vascular signals at adjacent areas, correlating with histopathological findings (Fig. 7A).

The system achieved single-scan coverage of a 25 mm field of view within 20 min, with complete specimen assessment requiring multi-position scans and image stitching. Its 7 mm effective penetration depth met the negative margin criteria. Key advantages included simultaneous visualization of vessels, lipid, and collagen/water signals, surpassing traditional structural imaging, such as US and X-ray. The 20-minute assessment outperformed frozen section analysis (30–40 min) and postoperative pathology (3–5 days), and it also eliminated the need for contrast agents, reducing the complexity of clinical operations. However, the 7 mm depth limitation hindered the evaluation of large or multifocal tumors, necessitating low-frequency probes (e.g., 3 MHz) or full-ring array designs. False positives arising from fibroglandular tissue (FGT), which exhibited reduced lipid signals and localized Hb enhancement, could be mitigated by extending the wavelength range (1200–1300 nm) to resolve collagen/water signals. In the future, studies with larger cohorts are required to validate universal applicability and refine intraoperative decision-making systems.

Carson et al. [99] developed a low-frequency hand-held photoacoustic probe prototype operating at 322 kHz, integrating a PVDF acoustic sensor and fiber-optic illumination. Optimized for macroscopic margin assessment, the probe leveraged lipid-content differences, where low lipid levels in tumor tissue generate weak signals, to evaluate surgical margins. Sensitivity was maximized for features 3.15–6.94 mm in size, suitable for detecting macroscopic structural anomalies. Phantom experiments validated its ability to identify low-absorption regions simulating tumors as small as 0.5 mm wide and 6 mm deep, with stable detection for features > 1 mm wide and an average width error of 1.36 mm. Limited sensor bandwidth (81 %) introduced negative artifacts, which can be mitigated by forced-zero postprocessing at the cost of slight overestimation of low-signal areas. Despite the artifacts induced by the ±42° scan angle, the probe achieved a higher signal-to-noise ratio (SNR) and enhanced lipid-tissue contrast compared to full-view systems. In ex vivo breast cancer specimens, the probe can detect strong signals from lipid-rich adipose tissue, though non-lipid structures (e.g., sutures) caused surface interference. In phantom experiments simulating positive margins, the probe successfully detected lipid layer notches (simulating tumor residues), but depth quantification remained restricted to superficial regions under 4 mm (Fig. 7B).

This technology's strengths lie in its non-destructive nature and real-time imaging potential. Compared to intraoperative devices such as MarginProbe®, it provides depth-resolved information (up to 6 mm) and continuous margin assessment. However, limitations include further miniaturization to adapt to surgical cavities, integration with optical tracking for freehand scanning, and potential improvements in surface localization through fusion with multispectral PA and ultrasound imaging. This study is the first to validate the feasibility of single-sensor hand-held photoacoustic technology based on lipid contrast for breast cancer margin assessment, offering a novel approach for intraoperative visualization of positive margins.

Wong et al. [100] developed a label-free ultraviolet photoacoustic microscopy (UV-PAM) system, which provides a fast label-free solution for intraoperative margin assessment in breast cancer. The technology leverages a pulsed ultraviolet laser light (266 nm) to selectively excite nucleic acids and proteins within cell nuclei. The system achieves a lateral resolution of 330 nm (single-cell nucleus visualization) and an axial resolution of 48 μm (depth-directed triple-somatosome stratification), which breaks through the diffraction limit of the traditional optical microscope. It uses Hilbert transform algorithm to enhance the axial signal resolution, which enables the three-dimensional spatial distribution of cell nuclei to be accurately rendered.

Technical validation demonstrated that UV-PAM has strong compatibility with gold-standard histopathology. Experimental data showed an imaging depth of 100 μm, covering approximately 20 times the thickness of conventional H&E-stained Sections (4–5 μm), thereby enabling continuous visualization from surface to subsurface structures. Although imaging of a 1 × 1 cm² area currently requires ∼7 h, parallel acquisition simulations using microlens array beam-splitting and a 64-channel ultrasound transducer suggest a theoretical acceleration factor exceeding 100-fold, reducing imaging time to clinically acceptable. Comparative studies revealed a strong correlation between UV-PAM images and H&E-stained sections in critical diagnostic metrics (Fig. 7C): in malignant regions, the system quantified nuclear atypia (40 % increase in average diameter, *p* < 0.01) and polarity disarray while detecting microsatellite foci (<200 μm) often missed by frozen section analysis, significantly improving intraoperative prediction of margin positivity.

### 3.2 Clinical translation and multimodal in vivo imaging

The clinical translation of PAI technology began in 2001 with the first prototype system, LOIS, which achieved in vivo tumor detection and localization in breast cancer patients [101], marking a pivotal transition from laboratory research to clinical application. Subsequent technological advancements led to the development of the LOIS-64 photoacoustic tomography system [102]. In a preliminary clinical trial involving 27 breast tumor patients, LOIS-64 successfully identified 18 out of 26 malignant lesions and 4 out of 8 benign lesions, demonstrating clinical feasibility. To further enhance practicality, the research team developed a hand-held laser optical imaging/grayscale ultrasonography (OA/US) multimodal imaging system, Imagio™ (developed by Seno Medical Instruments, USA, CE-certified) [103]. This system integrates dual-wavelength lasers (755 nm and 1064 nm) with a 128-element ultrasonic probe (5 MHz frequency), achieving a spatial resolution of 0.5 mm and a maximum penetration depth of 30 mm, significantly expanding intraoperative real-time imaging applications.

**Fig. 8:**
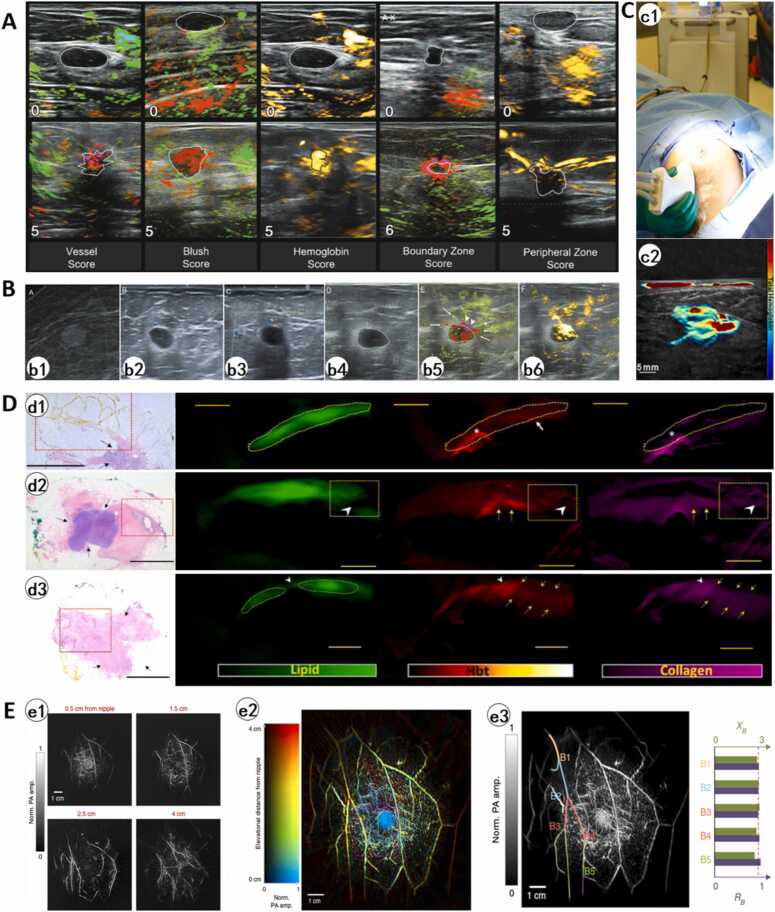
A. Reference key images demonstrate minimum and maximum scores for each OA feature. The top row is an example of the lowest score in each category and the bottom row is an example of the highest score in each category. Each OA feature score is described in detail in Table 3[104]. B. A grade II invasive ductal carcinoma that was upgraded at OA/US. b1, Mammography image. b2, US revealed a BI-RADS 4a oval, circumscribed, parallel hypoechoic mass with anterior thin capsule and posterior thick halo causing indistinct border. b3, Color Doppler image. b4, OA/US image. b5, OA/US imaging reveals intratumoral pleomorphic deoxygenated vessels with an anterior focal deoxygenated blush (arrowheads) and bilateral perpendicular boundary zone neovessels (arrows), demonstrating characteristic malignant angiogenesis patterns. b6, The OA/US total hemoglobin map shows markedly increased hemoglobin (the white segmentation line). The OA/US internal vessel score is 5. The internal hemoglobin score is 5, the boundary zone deoxygenated blush score is 6, and peripheral radiating vessel is 3. The estimator-derived probability of malignancy is 93 %. The mass was upgraded from BI-RADS 4a at gray-scale US and Doppler to 4c at OA/US [105]. C. The use of MSOT during the operation (c1). Visualization of breast tumor using MSOT (c2) [38]. D. d1. A Representative NACT specimen showing negative margins. HE stained image shows residual tumor (black arrows) that is at least 3 mm away from all margins (boxed area corresponds to PA signals). A continuous lipid, Hb and collagen signal (dotted area) is highly indicative of negative margins. High intensity blood and collagen signals (*) at the periphery of the excised tissue is due to orientation stitches. d2. Negative margin patterns. HE stained image shows residual tumor (black arrows) that is away from the margins, with surrounding fibrosis (tumor bed) extending closer to the margins (boxed area). There is thinning/disruption of the lipid layer noted (white arrowhead). This area contains collagen which is directly connected to the tumor bed (arrowheads). However, its collagen signal intensity is not equal or higher than the tumor bed (yellow arrows) and does not show increased vascularity. Hence, only 1 imaging PA criteria was met (collagen connectivity to tumor bed) and findings are supportive of negative margins. d3. Positive margin patterns. HE stained image shows residual tumor (black arrows) with some response to presurgical therapy. The tumor bed extends up to all margins grossly with DCIS extending very close to the inferior margin (1 mm) (red dotted box). The tumor cells (both invasive and in-situ) also extend very close to (1 mm or less than 1 mm away from) several other margins (anterior, superior, medial and lateral) microscopically. A disruption of the lipid layer is noted (white arrowheads) at the superior margin. This area contains high intensities of Hb and collagen (white arrowheads) with direct connectivity to the tumor bed (yellow arrows). The presences of all three PA imaging criteria for positive were met. Scale bars: HE staining, 10 mm, PA signals, 5 mm [110]. E. SBH-PACT of healthy breasts. e1. Vasculature in a breast of a healthy volunteer. Images at four depths are shown in increasing depth order from the nipple to the chest wall. e2. The same breast image with color-encoded depths. e3. A closeup view of the region outlined by the magenta dashed box in e2. e4. A selected vessel tree with five vessel bifurcations, labeled from B1 to B5 [63].

The clinical value of Imagio™ has been validated through large-scale trials. In the most extensive study to date, involving 2105 breast tumor patients [104], this system achieved a 40.8 % downgrade rate for benign masses, improving specificity from 28.1 % (ultrasound alone) to 43.0 %, while maintaining 96.0 % sensitivity for biopsy-confirmed malignancies. The negative likelihood ratio of 0.094 indicates that a negative OA/US result can reduce the maximum pretest probability of malignancy for BI-RADS 4B lesions from 17.8 % to 2 % (equivalent to BI-RADS 3), enabling safe downgrading of low-to-intermediate suspicion lesions and avoiding unnecessary biopsies. OA/US achieves a breakthrough in specificity (53 % improvement over conventional ultrasound) through functional-morphological fusion imaging while maintaining high sensitivity, establishing itself as an effective tool for distinguishing benign and malignant breast lesions. This capability is primarily based on its proprietary quantitative scoring system, which evaluates five key parameters (Fig. 8A): 1) the number of individually resolved vessels and their relative degree of deoxygenation (vessel score); 2) tumor blush, representing volume-averaged vessels too small to resolve individually (blush score); 3) the absolute hemoglobin concentration (hemoglobin score); 4) the quantity, oxygenation status, and vascular morphology of hemoglobin within the tumor boundary zone (boundary zone score); and 5) the hemoglobin characteristics and vessel architecture in peripheral regions (peripheral score). Each criterion was systematically scored on a 0–5 scale (Table 3). These metrics collectively capture both metabolic activity and microvascular patterns critical for precision diagnosis.

**Table 3:** OA/US feature scores: definitions and correlations with malignancy [104].

Score	Explanation	PPV
Internal features score		
**OA/US internal vascularity and deoxygenation (vessel score)**		
0	No internal vessels	24.6 % (315/1282)
1	Normal internal vessels without branches, red or green	19.6 % (590/3007)
2	Normal internal vessels with branches, mostly green	30.8 % (418/1355)
3	Internal speckle; green = red in amount and less red than background	44.2 % (1139/2576)
4	Internal speckle or signal; red > green and red > background	55.3 % (1749/3165)
5	Multiple internal red vessels	59.5 % (534/897)
**OA/US internal tumor blush and deoxygenation (blush score)**		
0	No internal vessels	24.9 % (82/342)
1	Minimal internal speckle, all green	19.1 % (392/2050)
2	Mild internal speckle; red = green and red + green < background	26.4 % (866/3286)
3	Mild internal speckle; red > green and both < background	42.0 % (884/2105)
4	Moderate internal speckle; red > green and red also > background	57.4 % (2264/3944)
5	Red blush almost fills lesion	46.3 % (257/555)
**OA/US relative internal Hgb (Hgb Score)**		
0	No internal Hgb	23.9 % (288/1205)
1	Minimal internal Hgb, less Hgb than background	26.9 % (1122/4164)
2	Minimal internal Hgb in discrete vessels, Hgb = background	38.4 % (613/1597)
3	Moderate internal Hgb in discrete vessels, Hgb = background	47.6 % (1213/2550)
4	Many large internal vessels containing Hgb amount > background	56.4 % (1396/2475)
5	Many large Hgb filled vessels almost fill central nidus of mass	38.8 % (113/291)
External features score		
**OA/US external boundary zone vascularity and deoxygenation (boundary zone score)**		
0	No capsular/boundary zone vessels	13.7 % (167/1223)
1	Normal capsular/BZ vessel(s) without branches (long, curved, parallel to capsule, not perpendicular to capsule)	10.1 % (287/28538)
2	Normal capsular/BZ vessel(s) with normal tapering acutely angled branches, mostly green	17.0 % (113/665)
3	Capsular/BZ speckle; green = red; red < background red	30.3 % (485/1600)
4	Capsular/BZ speckle; red > green; red > background red	47.3 % (1304/2757)
5	≥ 3 capsular/BZ red vessels, some perpendicular	78.0 % (1739/2230)
6	Boundary zone deoxygenated blush (complete or partial)	68.1 % (650/954)
**OA/US peripheral zone radiating vessels score (peripheral zone score)**		
0	No peripheral zone peritumoral vessels	18.3 % (362/1975)
1	1 or 2 peripheral zone feeding or draining vessels, at least one green, not in a radiating pattern	15.5 % (704/4544)
2	> 2 peripheral zone vessels, but random orientation, not radiating perpendicular to the surface of the mass	29.8 % (248/833)
3	1 or 2 peripheral zone radiating vessels	53.3 % (1072/2012)
4	> 2 peripheral zone radiating vessels on one side of the mass	77.5 % (905/1168)
5	> 2 peripheral zone radiating vessels on more than one side of the mass	83.1 % (1454/1750)

Note. PPV = positive predictive value.

Building upon these standardized criteria, a prospective multicenter study involving 209 patients with 215 BI-RADS 4 A/4B lesions further validated the clinical utility of OA/US [105]. This morpho-functional stratification method demonstrated Imagio™ benefits: 41 % of benign lesions were safely downgraded to BI-RADS 3/2, while 47 % of malignancies were appropriately upgraded to BI-RADS 4 C/5. Compared to conventional grayscale ultrasound, OA/US significantly enhanced specificity from 38.2 % to 47.2 % (*p* = 0.027), achieving simultaneous optimization of true-positive upgrades and false-positive downgrades (Fig. 8B). This system minimized unnecessary biopsies and provided molecular-functional insights for intraoperative decisions.

Based on this evidence, the FDA approved Imagio™ in January 2021 for adjunctive evaluation of BI-RADS 3–5 breast lesions, establishing it as the first regulatory-approved PA/US multimodal device [106], [107]. In the same year, the multispectral photoacoustic tomography (MSOT) Acuity Echo system developed by iThera Medical GmbH received the CE marking [108] in Europe. In 2022, Luxonus Inc.'s LME-01, a hemispherical array-based ultrasound transducer PAI platform, received approval for medical device use from Japan's Pharmaceuticals and Medical Devices Agency. Luxonus is conducting various clinical trials for the LME-01, including lymphatic and reproductive systems [109]. These approvals validate the safety and efficacy of PAI systems in a clinical context.

The publication of a pioneering phase 1 trial by McNally et al. represents the first intraoperative in vivo use of MSOT for identifying breast cancer during BCS (Fig. 8C) [38]. The results provide critical evidence on its safety and performance in a real-world surgical setting. The primary contribution of this work is its rigorous safety validation. The trial demonstrated that handheld MSOT imaging did not cause any adverse events or significant increases in skin temperature (remaining below 37℃) across patients of all Fitzpatrick skin types. This establishes a strong safety profile for the intraoperative application of this technology. Beyond safety, the study confirmed the functional efficacy of MSOT for tumor characterization. It successfully distinguished malignant from benign breast tissue in all 45 patients based on significantly elevated signals of deoxyhemoglobin, oxyhemoglobin, and total hemoglobin within tumors compared to contralateral normal tissue. This underscores the capability of MSOT to provide real-time, endogenous contrast-based visualization of cancers. Furthermore, the research highlighted the potential of MSOT in sentinel lymph node (SLN) mapping. MSOT preoperatively identified all three intraoperatively confirmed positive SLNs by detecting the optoacoustic signal of the injected dye isosulfan blue, without any false positives, pointing to its potential as a non-invasive adjunct to traditional SLN biopsy techniques.

This study serves as a crucial proof-of-concept, bridging the gap between preclinical promise and clinical utility. The findings underscore that MSOT can be safely and effectively integrated into the surgical workflow, providing real-time functional data that complements traditional ultrasound. It paves the way for future studies designed to directly evaluate the impact of MSOT-guided resection on achieving negative margins and reducing reoperation rates.

Furthermore, the latest research by Goh et al. prospectively evaluates the utility of MSOT inVision 512-ECHO system for intraoperative margin assessment in BCS following neoadjuvant chemotherapy (NACT) [110]. It successfully applies PAI in the ex vivo evaluation of lumpectomy specimens, enabling high-resolution visualization of key endogenous chromophores—namely lipids, collagen, and hemoglobin—at depths of up to 5 mm. A seminal contribution of this research is the development of a novel, systematic framework for interpreting ultrasound-guided photoacoustic (US-PA) images in the post-neoadjuvant chemotherapy (NACT) setting. This framework is built upon the analysis of three critical, interconnected patterns that align closely with the biomarkers discussed in this review. First, the relative signal intensity of collagen at the surgical margin compared to the tumor bed is assessed; an equal or greater intensity is considered suspicious for residual disease. Second, an increase in hemoglobin-derived vascularity at the margin, relative to the tumor bed or surrounding tissue, suggests tumor-associated angiogenesis. Third, the direct anatomical continuity of collagen or hemoglobin signals from the tumor bed to the surgical margin serves as a strong indicator of positive margins. These three PA imaging criteria have been well verified (Fig. 8D).

The clinical performance of this pattern-based approach was remarkable. US-PA achieved an overall diagnostic accuracy of 89.0 % across 100 assessed margins, with both sensitivity and negative predictive value (NPV) reaching 100 %. This perfect NPV is of paramount clinical utility, as it can provide surgeons with a high degree of confidence that a negative US-PA reading obviates the need for further re-excision. Consequently, this technology holds significant potential for minimizing re-operation rates and associated patient morbidity, directly addressing a key challenge in breast-conserving surgery.

In addition to commercially available systems, multiple investigational multimodal devices are also being applied to in vivo tumor margin detection. Carson et al. [111] introduced a novel intraoperative photoacoustic screening (iPAS) for lipid-weighted imaging of breast tumors, comparing its performance with preoperative dynamic contrast-enhanced MRI (DCE-MRI) and histopathology. The study enrolled 12 biopsy-confirmed invasive breast cancer patients, utilizing a portable iPAS scanner (930 nm wavelength) to perform rapid 6-minute lipid-weighted imaging and generate 3D images. Image reconstruction and optimization employed back-projection and iterative algorithms. Tumor volume and maximum diameter measurements from iPAS were statistically compared against DCE-MRI and histopathological results. Key findings demonstrated a strong correlation between iPAS and DCE-MRI in tumor volume measurements. While iPAS maximum tumor diameter significantly correlated with histopathology, a systematic overestimation of 8.3 mm was noted. Notably, iPAS maintained consistent tumor contrast regardless of breast density and offered radiation-free operation without requiring exogenous contrast agents. This technology presents a promising alternative for intraoperative tumor assessment, with potential applications in preoperative diagnosis and margin assessment.

The single-breath-hold photoacoustic computed tomography (SBH-PACT) system, developed by Lin et al. [63], achieves the multimodal integration of vascular density mapping and biomechanical property characterization. This innovative platform has a 512-element full-ring ultrasonic transducer array and a 1064 nm near-infrared laser source, optimized by a three-dimensional back-projection algorithm. It enables volumetric breast imaging within a 15-second breath-hold while eliminating respiratory motion artifacts. The system achieves a penetration depth of 4 cm in vivo and maintains a spatial resolution of 255 μm (a fourfold improvement over conventional MRI), allowing precise visualization of microvasculature as small as 258 μm in diameter (Fig. 8E). Beyond anatomical vascular imaging, its 10 Hz high-speed 2D imaging capability facilitates dynamic photoacoustic elastography, quantifying tissue stiffness variations to establish a dual-contrast diagnostic framework. In a prospective study involving seven breast cancer patients, SBH-PACT demonstrated exceptional clinical utility. It detected tumors occult to mammography, achieving twofold higher spatial resolution than conventional ultrasound. Notably, it accomplished this without breast compression, significantly enhancing patient comfort. A proprietary vascular skeletonization algorithm revealed tumor-associated vascular density ratios of 3.4 ± 0.99 compared to normal tissue (p < 0.001), correlating strongly with histopathologically confirmed angiogenesis. By integrating elastographic compensation which detected tumors based on their 50 % lower tissue compliance than normal parenchyma, this functional approach achieved 100 % sensitivity in diagnostically challenging cases. Furthermore, it resolved morphological details of DCIS lesions as small as 0.8 cm. Machine learning-driven tumor segmentation via vascular density heatmaps achieved automatic lesion localization (AUC = 0.90, specificity = 80 %), while arterial pulsation tracking (1.2 Hz cardiac rhythm) enabled arteriovenous differentiation, laying the groundwork for future blood oxygen saturation quantification. This study marks a significant milestone in clinical translation of PAI, offering a transformative paradigm for in vivo precision diagnostics and therapeutics in breast cancer, with emerging applications in intraoperative margin assessment.

### 3.3 Comparison of ex vivo and in vivo detection

In BCS, the precision of tumor margin assessment hinges on the ability to balance diagnostic accuracy with real-time clinical utility. Ex vivo techniques, conducted on excised specimens, prioritize molecular specificity and controlled analysis, while in vivo methods aim to deliver dynamic, intraoperative insights to the cavity without disrupting surgical workflows. This section systematically compares these two strategies' advantages, limitations, and clinical applicability within the context of PAI, elucidating their roles in advancing precision oncology and guiding technology development (summarized in Table 4)  .

**Table 4:** Comparative analysis of ex vivo vs. in vivo photoacoustic detection for margin assessment.

Aspect	Ex Vivo Detection	In Vivo Detection
Core Principle	Post-resection analysis of excised specimens under controlled, static conditions.	Real-time imaging within the surgical cavity during the procedure.
Primary Advantages	1. High analytical precision and specificity under controlled lab conditions. 2. Eliminates physiological interference (e.g., blood flow, motion). 3. Reduces direct patient risk from intraoperative probes or contrast agents.	1. Provides dynamic, intraoperative feedback for immediate surgical guidance. 2. High sensitivity for functional parameters (e.g., sO₂, viability). 3. Enables multimodal synergy (e.g., with US, fluorescence) for enhanced diagnostic accuracy.
Key Challenges	1. Lacks real-time capability; results are retrospective. 2. Post-excision artifacts (e.g., cell death, halted metabolism) compromise accuracy. 3. Limited spatial representativeness may miss heterogeneous marginal involvement.	1. Limited penetration depth due to optical scattering and acoustic impedance. 2. Signal attenuation from probe leakage or nonspecific efflux shortens observation windows. 3. Susceptible to motion artifacts from physiological cycles. 4. Potential long-term bioaccumulation risks of metal-based contrast agents. 5. Optical scattering artifacts distort image reconstruction. 6. Time-consuming scans for large areas prolong surgery.
Clinical Utility	Serves as a highly accurate, pathology-like validation tool, potentially replacing or supplementing frozen section analysis.	Functions as an intraoperative margin assessment or navigation tool for real-time guidance of resection boundaries and cavity assessment.
Technology Paradigm	Prioritizes analytical rigor and diagnostic specificity, often with higher resolution.	Emphasizes speed, integration into the surgical workflow, and real-time functional insight.

(1) **Advantages and Challenges of Ex Vivo Detection**

**Advantages:**

1. **High-Precision Analysis**: Ex vivo detection enables rigorous component analysis under controlled laboratory conditions, where metabolite concentrations are predefined and interactions minimized, facilitating unambiguous comparative studies.
2. **Eliminating Physiological Interference**: The absence of confounding factors such as body temperature and hemodynamic fluctuations ensures data reliability.
3. **Reduced Patient Risk**: Because it is performed outside the body, it avoids the potential adverse effects of intraoperative probes or contrast agents on patients.

**Challenges:**

1. **Lack of Real-Time Capability:** Samples must be excised before analysis, preventing dynamic monitoring.
2. **Post-Excision Artifacts:** Tissue excision alters natural states through processes like cell death and halted metabolic activity, compromising accuracy.
3. **Limited Spatial Representativeness:** Sampled regions may fail to represent tumor heterogeneity fully, increasing residual cancer risks at the margins.

(2) **Advantages and Challenges of In Vivo Detection**

**Advantages:**

1. **Real-Time Dynamic Imaging:** Captures intraoperative biological dynamics (e.g., blood oxygenation shifts), enabling immediate surgical decisions.
2. **High Sensitivity:** Modalities like photoacoustic computed tomography (PACT) achieve sub-millimolar sensitivity for applications such as stem cell tracking and viability assessment.
3. **Multimodal Synergy:** Integration with complementary techniques (e.g., fluorescence, ultrasound) enhances diagnostic accuracy and information depth.

**Challenges:**

1. **Limited Penetration Depth:** Visible light penetration is restricted by photon absorption and scattering, while acoustic wave transmission is impeded in bone or air-filled cavities. Enhancing transducer sensitivity (e.g., low-frequency arrays) is critical for deep-tissue imaging.
2. **Signal Attenuation:** Probe leakage or nonspecific cellular efflux shortens effective observation windows. Directly labeled tracers, though bioorthogonal, may lose accuracy during cellular imaging.
3. **Motion Artifacts:** Cardiac/respiratory cycles or involuntary movements degrade image stability, necessitating motion-correction algorithms.
4. **Long-Term Bioaccumulation Risks:** Metal-based nanoparticles (e.g., gold, copper) pose potential toxicity concerns.
5. **Optical Scattering Artifacts:** Wavelength-dependent light scattering distorts image reconstruction, requiring multispectral compensation strategies.
6. **Time-Consuming Scans:** Large-area imaging prolongs surgical duration, demanding rapid-scan protocols (e.g., parallelized transducer arrays).

The dichotomy between ex vivo and in vivo detection underscores a fundamental tension in oncologic imaging: the pursuit of analytical rigor versus the demand for real-time adaptability. While ex vivo methods excel in resolving molecular heterogeneity under controlled conditions, their delayed feedback and spatial limitations hinder intraoperative utility. Conversely, in vivo PAI dynamically bridges functional and anatomical insights but grapples with the challenges of penetration depth, motion artifacts, and biocompatibility. Emerging solutions, such as hybrid systems integrating ex vivo validation with in vivo evaluation, or nanoparticle probes optimized for specificity and safety, promise to synergize these approaches. By leveraging the strengths of each modality, next-generation PAI technologies may redefine intraoperative decision-making, thereby minimizing residual disease risks while preserving patient quality of life.

## 4 Challenges, clinical translation, and future directions

PAI combines high spatial resolution of ultrasound with superior optical absorption contrast, all without ionizing radiation. It provides highly sensitive information on blood oxygen metabolism and vascular topological topology, which are critical for delineating tumor boundaries. However, the clinical translation of PAI faces multifaceted challenges that must be addressed to meet rigorous surgical demands. This section critically examines the technical challenges, documents the pivotal progress in clinical standardization, and envisions the trajectory toward intelligent, integrated systems that can redefine precision oncology.

### 4.1 Technical hurdles in enhancing intraoperative applications

The efficacy of PAI as an intraoperative tool is contingent upon overcoming fundamental physical and technical limitations that impact its accuracy and reliability in the dynamic surgical environment.

A primary challenge is the limited tissue penetration depth, which is crucial for assessing deep resection margins. Photon scattering and absorption in biological tissues cause exponential signal attenuation, particularly in dense breasts, where the signal-to-noise ratio (SNR) for lesions deeper than 30 mm deteriorates significantly. This issue is exacerbated in large-volume breast imaging, where spatial heterogeneity of light distribution and complex acoustic wave propagation (e.g., speed variations, dispersion, and attenuation) lead to phase shifts and amplitude distortions, degrading image reconstruction quality [112]. Future advancements must focus on developing low-frequency ultrasound transducers and sophisticated image reconstruction algorithms that compensate for acoustic attenuation to improve deep-tissue imaging fidelity.

Furthermore, multi-physical coupling interference poses a significant challenge for accurate spectral unmixing, a process vital for differentiating tumor-specific biomarkers (like hypoxia) from surrounding healthy tissue [113]. Nonlinear optical absorption responses and nonlocal effects such as lateral photon diffusion can distort quantitative readings of blood oxygen saturation (sO₂) or contrast agent concentration [114]. For intraoperative margin assessment, this necessitates the integration of computationally efficient, real-time light transport models, like accelerated Monte Carlo simulations, into the imaging system to correct for fluence variations and ensure the fidelity provided to the surgeon.

### 4.2 Integration with surgical workflow and decision-support

Beyond physics-based challenges, the practical integration of PAI into the surgical workflow presents its own set of requirements, centered on hardware performance and the critical trade-off between resolution and imaging depth.

The translation of PAI into the operating room hinges on overcoming hardware performance bottlenecks. Current ultrasound transducers with limited bandwidth and high noise-equivalent pressure hinder the detection of weak signals from subtle pathological changes at the margin, which are often the hallmark of residual disease. There is an urgent need for high-sensitivity, wide-bandwidth transducers that can be integrated into compact, handheld, and sterilizable probes compatible with the sterile surgical field.

Additionally, the inherent resolution-depth trade-off constrains detection precision. As imaging depth increases, high-frequency ultrasound components attenuate, reducing axial resolution to millimeter scales [115]. Emerging multi-scale imaging paradigms, which combine deep-penetrating PACT for an overview with high-resolution surface scanning via PAM for detailed margin inspection, promise a comprehensive solution for cavity assessment.

### 4.3 Standardization and clinical adoption: building the pathway to practice

Despite these challenges, the clinical translation of PAI has achieved critical milestones. A critical step has been the development of standardized protocols to ensure data reliability and system interoperability, which are crucial for multi-center trials and clinical translation.

A pivotal milestone was reached in 2023. The International Photoacoustic Standardization Consortium (IPASC) formally incorporated PAI into the Digital Imaging and Communications in Medicine (DICOM) standard [48]. This resolves long-standing data interoperability challenges, enabling seamless integration of PAI data into hospital Picture Archiving and Communication Systems (PACS) and facilitating comparison with MRI and ultrasound. Concurrently, the American Medical Association (AMA) introduced a provisional Current Procedural Terminology (CPT) code (0857 T) for photoacoustic-ultrasound imaging, activated in 2024 [48]. This coding reform is instrumental for standardized billing and insurance reimbursement, which is crucial for supporting widespread clinical adoption and enabling structured, large-scale multicenter trials to validate the impact of PAI margin assessment on surgical outcomes [8].

### 4.4 Future directions: toward intelligent integrated navigation systems

Looking forward, the future of PAI in BCS lies in the evolution from an imaging tool to an intelligent, integrated navigation system. This paradigm shift will be driven by the convergence of advanced hardware, artificial intelligence (AI), and seamless integration with surgical platforms.

The core of this evolution is AI-powered decision support. Deep learning algorithms can be trained to automatically analyze the multi-parametric data from PAI—including sO₂ maps, vascular density, and biomarker distribution—to generate intuitive, color-coded heatmaps that predict margin status in real-time. This offers dynamic feedback on the residual distance to the tumor margin, empowering surgeons to adjust their dissection plane dynamically for a more guided surgical approach.

Furthermore, these intelligent systems are poised for integration with robotic surgical platforms. PAI could provide real-time, intraoperative tissue characterization that guides robotic instruments, enabling highly precise, biomarker-defined resection boundaries. This closed-loop navigation system would represent the ultimate fulfillment of PAI's potential, minimizing residual disease risks while maximizing healthy tissue preservation. By focusing next-generation developments on intelligence, integration, and speed, PAI is poised to revolutionize the paradigm of precision oncology in breast cancer surgery, directly improving patient outcomes and quality of life.

## 5 Conclusion

Photoacoustic imaging (PAI) holds immense promise in breast-conserving surgery (BCS) for breast cancer. During intraoperative tumor margin assessment, its unique capability to provide real-time, high-contrast tissue characterization enables precise tumor resection, minimizing residual cancer risks while preserving healthy breast tissue integrity. However, the widespread clinical implementation of PAI faces significant challenges, including imaging depth and sensitivity limitations, which must be optimized to meet rigorous surgical demands. Furthermore, establishing standardized protocols and validation criteria is critical to ensuring consistent and reliable clinical application. As these challenges are systematically addressed, PAI is poised to revolutionize BCS by improving surgical precision, reducing tumor residue and reoperation rates, and ultimately enhancing patient prognosis and quality of life. This advancement will invigorate the evolution of precision oncology in breast cancer surgery, setting a new benchmark for minimally invasive, patient-centric therapeutic strategies.

## CRediT authorship contribution statement

**Guangwei Chen:** Writing – review & editing, Investigation. **Jiayu Wang:** Writing – review & editing, Data curation, Conceptualization. **Runqi Zhao:** Writing – review & editing, Data curation. **Ye Zhang:** Writing – review & editing, Validation. **Yiqiong Zheng:** Writing – review & editing, Writing – original draft, Formal analysis, Data curation, Conceptualization. **Zhijie Luo:** Writing – review & editing, Writing – original draft, Visualization, Formal analysis, Data curation, Conceptualization. **Wenye Gong:** Writing – review & editing, Investigation, Data curation, Conceptualization. **Ruixi Sun:** Writing – review & editing, Investigation, Formal analysis, Conceptualization. **Xiru Li:** Supervision, Resources, Project administration, Funding acquisition. **Fei Gao:** Supervision, Project administration, Methodology. **Daohuai Jiang:** Supervision, Project administration, Methodology.

## Declaration of Competing Interest

The authors declare the following financial interests/personal relationships which may be considered as potential competing interests: Yiqiong Zheng reports financial support was provided by National Natural Science Foundation of China. Given his role as associate editors of Photoacoustics, Fei Gao had no involvement in the peer review of this article and had no access to information regarding its peer review. Full responsibility for the editorial process for this article was delegated to another journal editor. If there are other authors, they declare that they have no known competing financial interests or personal relationships that could have appeared to influence the work reported in this paper.

## Data Availability

No data was used for the research described in the article.
